# Individual cristae within the same mitochondrion display different membrane potentials and are functionally independent

**DOI:** 10.15252/embj.2018101056

**Published:** 2019-10-14

**Authors:** Dane M Wolf, Mayuko Segawa, Arun Kumar Kondadi, Ruchika Anand, Sean T Bailey, Andreas S Reichert, Alexander M van der Bliek, David B Shackelford, Marc Liesa, Orian S Shirihai

**Affiliations:** ^1^ Department of Medicine (Endocrinology) Department of Molecular and Medical Pharmacology David Geffen School of Medicine University of California Los Angeles CA USA; ^2^ Graduate Program in Nutrition and Metabolism Graduate Medical Sciences Boston University School of Medicine Boston MA USA; ^3^ Institute of Biochemistry and Molecular Biology I Medical Faculty Heinrich Heine University Düsseldorf Düsseldorf Germany; ^4^ Department of Pulmonary and Critical Care Medicine David Geffen School of Medicine University of California Los Angeles CA USA; ^5^ Jonsson Comprehensive Cancer Center David Geffen School of Medicine University of California Los Angeles CA USA; ^6^ Lineberger Comprehensive Cancer Center University of North Carolina at Chapel Hill Chapel Hill NC USA; ^7^ Molecular Biology Institute at UCLA Los Angeles CA USA; ^8^ Department of Biological Chemistry David Geffen School of Medicine at UCLA Los Angeles CA USA

**Keywords:** crista junction, cristae, membrane potential, MICOS complex, Opa1, Membrane & Intracellular Transport

## Abstract

The mitochondrial membrane potential (ΔΨ_m_) is the main driver of oxidative phosphorylation (OXPHOS). The inner mitochondrial membrane (IMM), consisting of cristae and inner boundary membranes (IBM), is considered to carry a uniform ΔΨ_m_. However, sequestration of OXPHOS components in cristae membranes necessitates a re‐examination of the equipotential representation of the IMM. We developed an approach to monitor ΔΨ_m_ at the resolution of individual cristae. We found that the IMM was divided into segments with distinct ΔΨ_m_, corresponding to cristae and IBM. ΔΨ_m_ was higher at cristae compared to IBM. Treatment with oligomycin increased, whereas FCCP decreased, ΔΨ_m_ heterogeneity along the IMM. Impairment of cristae structure through deletion of MICOS‐complex components or Opa1 diminished this intramitochondrial heterogeneity of ΔΨ_m_. Lastly, we determined that different cristae within the individual mitochondrion can have disparate membrane potentials and that interventions causing acute depolarization may affect some cristae while sparing others. Altogether, our data support a new model in which cristae within the same mitochondrion behave as independent bioenergetic units, preventing the failure of specific cristae from spreading dysfunction to the rest.

## Introduction

Mitochondria utilize nutrients and molecular oxygen to generate a membrane potential (ΔΨ_m_) across the inner mitochondrial membrane (IMM). The energy available for ATP synthesis is directly derived from ΔΨ_m_ (Mitchell, 1961; Mitchell & Moyle, 1969); therefore, depolarization directly translates to decreased energy availability for ATP synthesis.

Classical studies suggested that the ΔΨ_m_ was homogeneous along the IMM. Data supporting the equipotential model are (i) mitochondria labeled with ΔΨ_m_‐dependent dyes show a homogeneous signal along a single mitochondrion visualized under a low resolution microscope, indicating that the ΔΨ_m_ is likewise homogeneous all along the organelle (Amchenkova *et al*, 1988; Skulachev, 2001); and (ii) an elongated mitochondrion stained with a ΔΨ_m_‐dependent dye appears to instantaneously lose its ΔΨ_m_ following laser‐induced damage to a small (≤ 0.5 μm^2^) region, suggesting that a mitochondrial filament is analogous to a power cable, where, if one part is compromised, the voltage will simultaneously collapse across its entire length (Amchenkova *et al*, 1988; Skulachev, 2001; Glancy *et al*, 2015). These conclusions were drawn after imaging mitochondria with ΔΨ_m_‐dependent dyes performed with light microscopes lacking sufficient spatial resolution to visualize the ultrastructure of the IMM. Furthermore, previous studies lacked the temporal resolution to determine whether laser‐induced depolarization leads to an instantaneous collapse of ΔΨ_m_ across the whole organelle.

The IMM consists of subcompartments called cristae and inner boundary membrane (IBM) (Palade, 1953). Cristae are invaginations protruding into the mitochondrial matrix, whereas the IBM runs parallel to the outer mitochondrial membrane (OMM). Cristae and IBM are connected via narrow tubular or slit‐like structures, known as crista junctions (CJs). In recent years, studies show that components of the electron transport chain (ETC) are confined to the lateral surfaces of the cristae rather than equally distributed along the IMM (Vogel *et al*, 2006; Wilkens *et al*, 2013). Moreover, dimers of *F*_1_ *F*_0_ ATP Synthase assemble in rows along the edges of the cristae (Dudkina *et al*, 2005; Strauss *et al*, 2008; Davies *et al*, 2011). The CJs can be kept in a closed state by oligomers of the inner‐membrane dynamin‐like GTPase, OPA1 (Frezza *et al*, 2006; Pham *et al*, 2016), as well as components of the mitochondrial contact site and cristae organizing system (MICOS complex) (John *et al*, 2005; Rabl *et al*, 2009; Barrera *et al*, 2016; Glytsou *et al*, 2016).

These findings provide a conceptual framework, where protons pumped by the ETC across the cristae membrane appear first in the cristae lumen (Busch *et al*, 2013; Pham *et al*, 2016). However, unless the ΔΨ of the crista membrane is kept more negative compared to its neighboring IBM, protons would not remain in the cristae. This consideration implies that differences in ΔΨ_m_ would exist between the cristae membrane and the IBM.

To establish whether the distribution and structural properties of OXPHOS complexes are functionally significant, it would be critical to directly visualize and quantify the ΔΨ_m_ in relation to the IMM in living cells. If the ΔΨ_m_ is uniform from one end of a mitochondrion to another, the ΔΨ_m_ would be equal at any point along the IMM, supporting the equipotential model. If, however, the ΔΨ_m_ stems from cristae functioning as independent and heterogeneous compartments, the ΔΨ_m_ would vary substantially along the IMM—between cristae and IBM, as well as between different cristae. Testing such hypotheses, nonetheless, has remained virtually unfeasible, because the only way to resolve the IMM has been with the electron microscope, which requires freezing or fixation of mitochondria and therefore precludes any direct measurement of structure and ΔΨ_m_.

To overcome this limitation, we developed a novel approach for imaging the IMM at high spatiotemporal resolution in living cells, using the LSM880 with Airyscan as well as STED microscopy. Staining active mitochondria with various dyes, we verified that we can resolve cristae from IBM. We then used various ΔΨ_m_‐dependent dyes to explore how the intricate architecture of the IMM relates to the most basic mitochondrial function—the ΔΨ_m_ generated by the electrochemical gradient of protons.

## Results

### Development of an Airyscan‐based approach to resolve cristae and IBM in living cells

**Figure 1:**
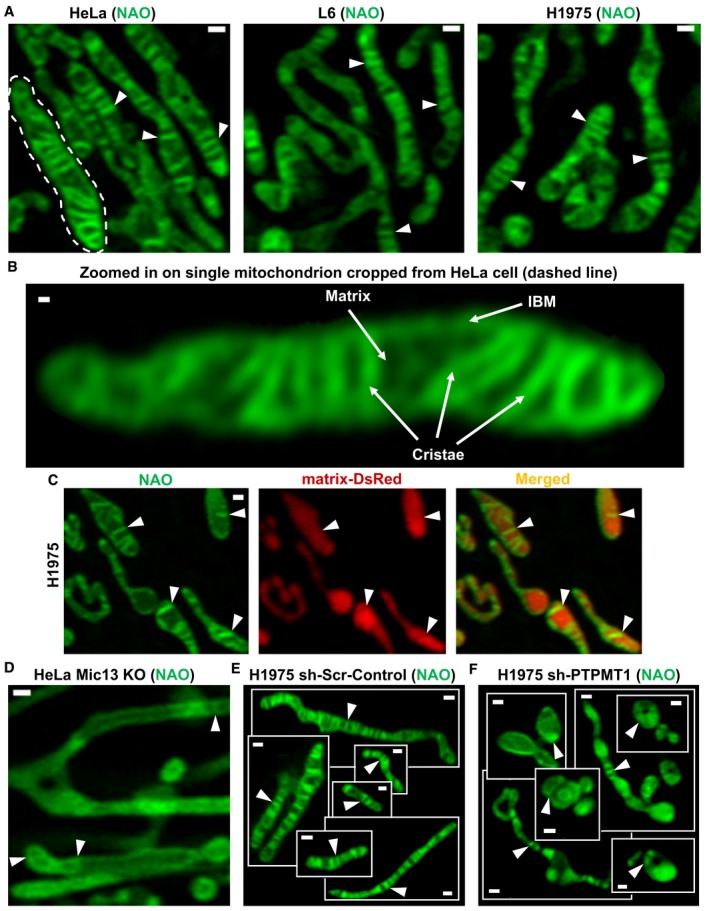
High‐resolution fluorescence imaging using Airyscan resolves the inner mitochondrial membrane (IMM) structure in live cells High‐resolution imaging of mitochondria in live cells using the Airyscan module of Zeiss LSM880 confocal microscope.
AImages of IMM in living HeLa, L6, and H1975 cells, stained with 10‐*N*‐nonyl acridine orange (NAO). NAO preferentially binds phospholipids in the IMM, such as cardiolipin. Arrowheads indicate cristae in the IMM. Scale bar = 500 nm. *N* ≥ 3 independent experiments for each cell type.BMitochondrion cropped from HeLa cell shown in (A) (dashed line) and zoomed in to show cristae, inner boundary membrane (IBM), and matrix (arrows). Scale bar = 100 nm.CH1975 cells transduced with matrix‐targeted DsRed and stained with NAO. Matrix‐targeted DsRed differentiates matrix from cristae stained with NAO. Arrowheads point to cristae. Scale bar = 500 nm. *N* = 1 independent experiment.DThe structure of the IMM in Mic13‐KO cells (HeLa), stained with NAO. The number of cristae is decreased in Mic13‐KO compared to control cells shown in panel (A), labeled with arrowheads. Scale bar = 500 nm. *N* = 3 independent experiments.E, FLive‐cell imaging of the IMM in control and PTPMT1 KD H1975 cells, a model of cardiolipin deficiency. (E) A gallery of mitochondria from various control H1975 cells expressing scrambled shRNA and stained with NAO, showing cristae (arrowheads). Scale bars = 500 nm. *N* = 3 independent experiments. (F) A gallery of mitochondria from various H1975 cells expressing PTPMT1 shRNA and stained with NAO. Note the derangement of the ultrastructure (arrowheads). Scale bars = 500 nm. *N* = 3 independent experiments.

**Figure EV1:**
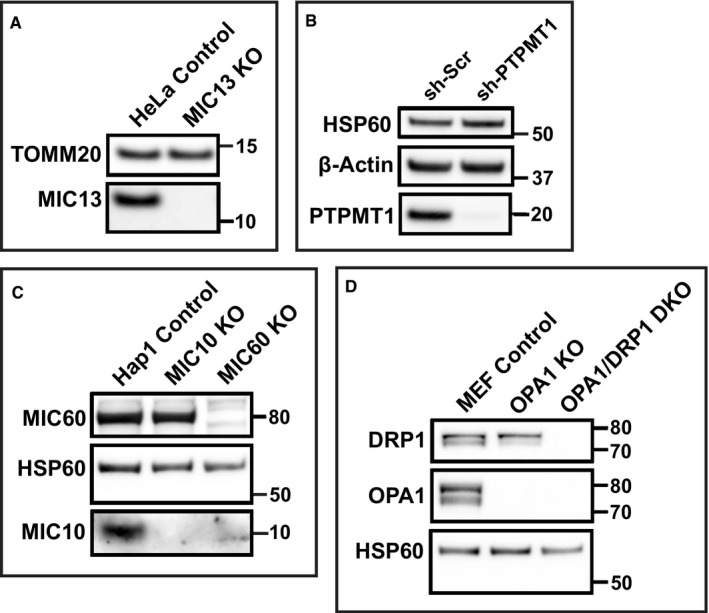
Validation of KO and KD approaches of Cristae structure and CJ modulators Representative image of Western blot, showing deletion of MIC13 in HeLa cells. *N* = 3 independent experiments.Representative image of Western blot, showing deletion of PTPMT1 in H1975 cells. *N* = 3 independent experiments.Representative image of Western blot, showing deletion of MIC60 and MIC10 in Hap1 cells. *N* = 3 independent experiments.Representative image of Western blot, showing Opa1 KO and Opa1&Drp1 DKO in MEFs. *N* = 3 independent experiments. Source data are available online for this figure.

Previous studies show that components of OXPHOS are unevenly distributed between the cristae and IBM (Vogel *et al*, 2006; Wilkens *et al*, 2013), suggesting the possibility of ΔΨ_m_ heterogeneity along the IMM within a single mitochondrion. To develop an approach for the imaging of ΔΨ_m_ associated with cristae and IBM, we first sought to determine whether we could resolve the compartmentalization of the IMM in living cells. To address this question, we incubated various cell types with 10‐*N*‐nonyl acridine orange (NAO), a fluorescent probe that preferentially binds cardiolipin but also shows some affinity for other phospholipids found in mitochondria, such as phosphatidylethanolamine (PE) and phosphatidylinositol (PI) (Leung *et al*, 2014). Imaging mitochondria from living HeLa, L6, and H1975 cells with the LSM880 equipped with Airyscan technology, we resolved intramitochondrial structures, typically perpendicular to the long axis of the mitochondrion, resembling cristae, as observed in electron micrographs (Fig 1A). Accordingly, the high resolution of the Airyscan‐based microscopy allowed separation of cristae structures, IBM, as well as dimmer regions, appearing to be matrix (Fig 1B). To verify that they were matrix, we used matrix‐targeted DsRed to label the matrix in H1975 cells and stained their IMM with NAO (Fig 1C). Airyscan imaging confirmed that the dimmer regions of NAO fluorescence within the mitochondria showed the strongest matrix‐DsRed signal and vice versa (arrowheads). To confirm that we correctly identified the cristae structures in cells stained with NAO, we tested whether the pattern of NAO labeling was changed in cells with disrupted cristae structure. As a model, we used HeLa cells with Crispr/Cas9‐mediated KO of Mic13 (Fig EV1A), which destabilizes CJs and disrupts cristae structure (Fig 1D) (Anand *et al*, 2016; Guarani *et al*, 2015). Compared to control HeLa (Fig 1A and B), Mic13‐KO mitochondria showed a substantial decrease in the number of perpendicular structures, supporting their identification as cristae. As a second model of cristae perturbation, we examined H1975 cells with stable KD of PTPMT1 through lentiviral transduction encoding shRNA (Fig EV1B). PTPMT1 is a mitochondrial phosphatase, essential for biosynthesis of phosphatidylglycerol, a precursor of cardiolipin. Deletion of PTPMT1 has been shown to result in severe derangement of the IMM (Zhang *et al*, 2011). Our Airyscan imaging of sh‐Scramble (sh‐Scr) control mitochondria shows structures closely resembling normal cristae (Fig 1E), whereas sh‐PTPMT1 mitochondria display a variety of deformed structures (Fig 1F) analogous to cristae perturbations that were previously observed in electron micrographs of PTPMT1‐deficient models (Zhang *et al*, 2011). Overall, these data demonstrate that Airyscan technology can resolve mitochondrial ultrastructure in living cells. We subsequently used this approach to measure ΔΨ_m_ at the different compartments along the IMM and determine the level of heterogeneity in ΔΨ_m_ within the individual mitochondrion.

### ΔΨ_m_‐dependent dyes colocalize most strongly with cristae

**Figure 2:**
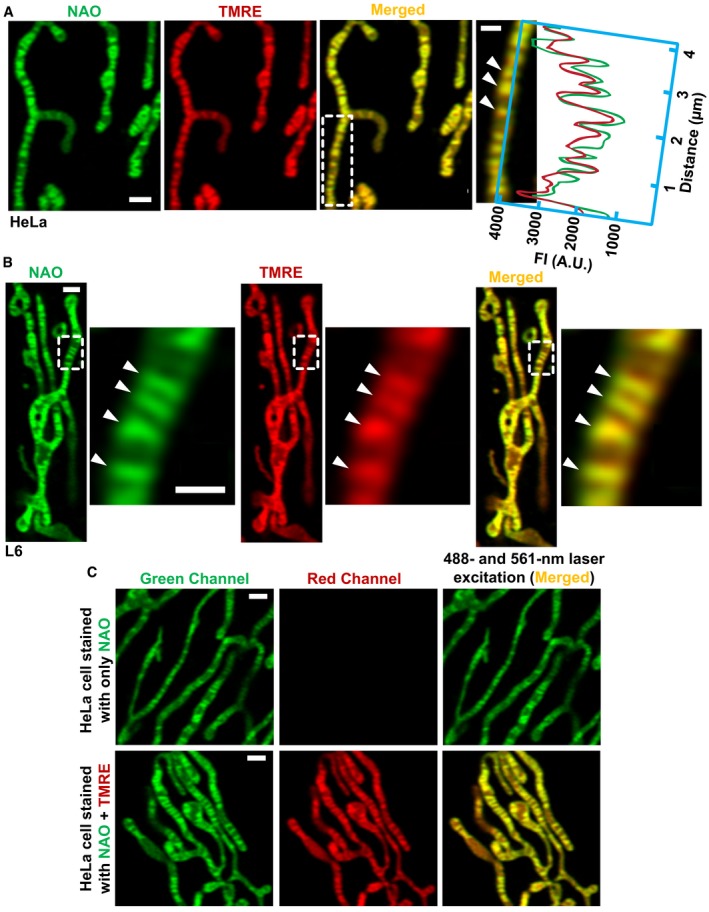
The ΔΨ_m_‐sensitive dye, TMRE, partitions to cristae stained with NAO High‐resolution imaging of mitochondria in live cells using the Airyscan module of Zeiss LSM880 confocal microscope.
Mitochondria from HeLa cell, co‐stained with NAO and TMRE. Area from dashed white box, zoomed to right, shows the red and green intensities of TMRE and NAO colocalized (arrowheads). Scale bars = 500 nm. *N* = 3 independent experiments.Mitochondria in L6 myoblast, co‐stained with NAO and TMRE. Area from dashed white box, zoomed to right, shows colocalizing NAO and TMRE at the cristae membrane (arrowheads). Scale bars = 500 nm. *N* = 3 independent experiments.Mitochondria in HeLa cells, stained with NAO alone (top row) vs. NAO + TMRE (bottom row), and simultaneously excited with 488‐ and 561‐nm lasers. Note that, mitochondria stained with NAO alone do not emit noticeable fluorescence in the red channel; only after adding TMRE does strong signal appear in the red channel, showing negligible bleed‐through. Scale bars = 500 nm. *N* = 2 independent experiments.

The power‐cable model of the ΔΨ_m_ assumes an electrical continuity along the IMM without any electrical resistance (Skulachev, 2001). However, the possibility of heterogeneity in ΔΨ_m_ along the IMM could not be investigated until now. To image ΔΨ_m_ along the IMM, we stained HeLa cells with NAO and TMRE (Farkas *et al*, 1989; Loew *et al*, 1993). Remarkably, TMRE appeared to align with the IMM in a non‐homogeneous manner, where the most‐intense TMRE signal colocalized with NAO at cristae (Fig 2A). To substantiate this observation, we looked at L6 (rat myoblast) cells, which presented the same heterogeneous pattern as HeLa cells (Fig 2B).

When excited by a 488‐nm laser, NAO has an emission spectrum that is limited to green wavelengths. However, in these experiments, we needed to excite NAO with the 488‐nm laser while simultaneously exciting TMRE with the 561‐nm laser, resulting in NAO being exposed to both 488‐ and 561‐nm lasers. The observed alignment of TMRE signal with the membrane staining by NAO raised the possibility that exciting NAO with the 561‐nm laser could result in red light emission and thus be wrongly detected as TMRE. To address this possibility, we imaged cells stained with NAO alone and excited simultaneously with 488‐ (NAO_EX_) and 561‐nm (TMRE_EX_) lasers (Fig 2C, top row). We found that emission of NAO after excitation with the 561‐nm laser (TMRE_EX_) was undetectable. After adding TMRE to the cells, initially stained with NAO alone, and then exciting with the 561‐nm laser using the same power, we observed the appearance of strong signal in the red channel, with most‐intense pixels colocalizing with NAO at cristae (Fig 2C, bottom row).

**Figure EV2:**
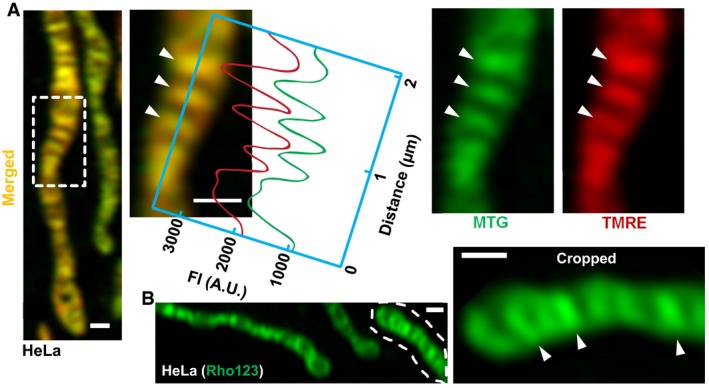
Membrane potential dyes, TMRE and Rho123, are concentrated at the cristae membranes in a heterogeneous pattern Mitochondria in HeLa cells co‐stained with MTG and TMRE. Zoomed‐in region from dashed white box highlights colocalization of dyes, showing that signal intensities vary together across the long axis (green and red lines); arrowheads indicate cristae membranes. Scale bar = 500 nm. *N* = 3 independent experiments.Mitochondria in HeLa cell stained with ΔΨ_m_‐dependent dye, Rho123; zoomed‐in mitochondrion cropped from dashed line highlights more intense signal at cristae (arrowheads). Scale bar = 500 nm. *N* = 3 independent experiments.

To further validate our findings with TMRE and NAO, we used two additional dyes, MitoTracker Green (MTG) and Rhodamine123 (Rho123). MTG covalently binds to various proteins embedded in the cristae membrane and, as such, is considered a ΔΨ_m_‐independent dye, although its initial sequestration in mitochondria depends on ΔΨ_m_ (Presley *et al*, 2003). Rho123 is a ΔΨ_m_ probe, which partitions to mitochondria in a transient way, indicating changes to ΔΨ_m_ (Ward *et al*, 2000; Duchen, 2004). We found that MTG colocalized with TMRE, showing a similar heterogeneous pattern (Fig EV2A). Then, we examined the partitioning of Rho123 and found that it shows the most‐intense signal associated with cristae (Fig EV2B).

**Figure 3:**
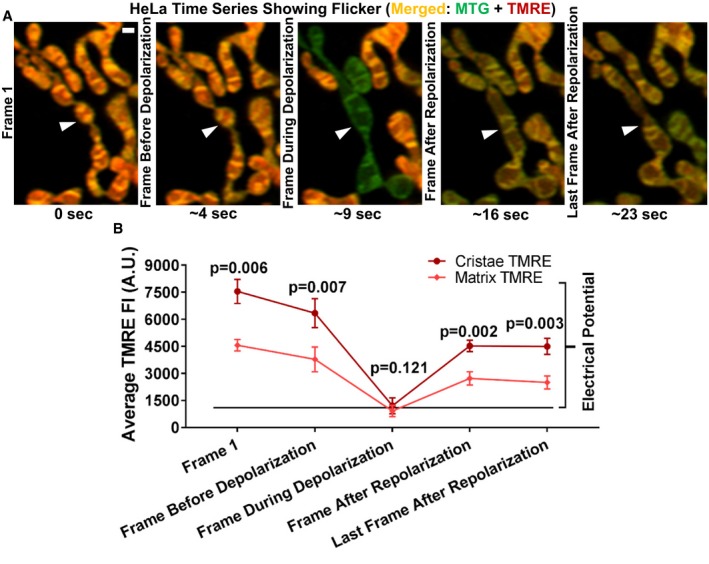
Validation that TMRE partitioning to cristae is ΔΨ_m_‐dependent Time‐lapse Airyscan imaging of ΔΨ_m_ in living HeLa cell, co‐stained with MTG (ΔΨ_m_‐insensitive after loading) and TMRE (ΔΨ_m_ sensitive). Arrowhead points to a flickering event where a mitochondrion depolarizes (˜ 9 s) and repolarizes (˜ 16 s), showing that heterogeneous signal from TMRE (but not MTG) disappears and reappears. Scale bar = 500 nm. *N* = 4 independent experiments.Quantification of (A). Plot shows average TMRE fluorescence intensity (FI) of cristae (dark red line) vs. matrix (light red line) during the time series. The drop in TMRE FI during the depolarization phase of the flickering is the ΔΨ_m_‐sensitive component of the TMRE signal. The remaining TMRE FI during depolarization can be considered as the ΔΨ_m_‐insensitive portion of TMRE signal. Note that, the remaining TMRE FI after depolarization at the cristae and matrix is approximately identical, indicating that differences in TMRE FI between the cristae and matrix prior to depolarization are derived from differences in ΔΨ_m_. *N* = 4 independent experiments.Data information: Data were analyzed with 2‐tailed Student's *t*‐tests, and *P* values < 0.05 were considered statistically significant. Specific *P* values are indicated in the figure. Error bars indicate SEM.

To further verify that the staining patterns of TMRE depend on ΔΨ_m_, we used a previously described method to influence ΔΨ_m_: Continuous exposure of TMRE‐stained mitochondria to the 488‐nm laser results in robust and rapid depolarization and repolarization, a phenomenon known as flickering (Duchen *et al*, 1998). We reasoned that the smaller portion of TMRE bound to the membrane in a ΔΨ_m_‐independent manner would remain during the flickering event and reveal the level of noise. Moreover, if the differences in TMRE fluorescence intensity (FI) between cristae and IBM depend on ΔΨ_m_, we would expect that the differences between the brightest and dimmest pixels would markedly decrease during depolarization. Conversely, following repolarization, we would expect these differences in pixel intensities to return. Figure 3A shows an example of a mitochondrion from a HeLa cell stained with MTG and TMRE (arrowheads), where we initially observed the heterogeneous patterns of TMRE; however, at ~ 9 s, the mitochondrion depolarized, and the heterogeneous staining pattern of TMRE was lost. Notably, at ~ 16 s, this mitochondrion repolarized and exhibited nearly the same TMRE heterogeneity as before the depolarization. Quantification of the changes in ΔΨ_m_ during the flickering phenomenon demonstrates that ~ 85% of TMRE signal was lost during depolarization (Fig 3B). Moreover, the differences between the brightest and dimmest areas (cristae and matrix, respectively) are attenuated during transient depolarization but are reestablished following restoration of ΔΨ_m_. These data support that the heterogeneous staining patterns of the TMRE are due to differences in ΔΨ_m_.

### Quantification of ΔΨ_m_ differences between cristae and IBM

**Figure 4:**
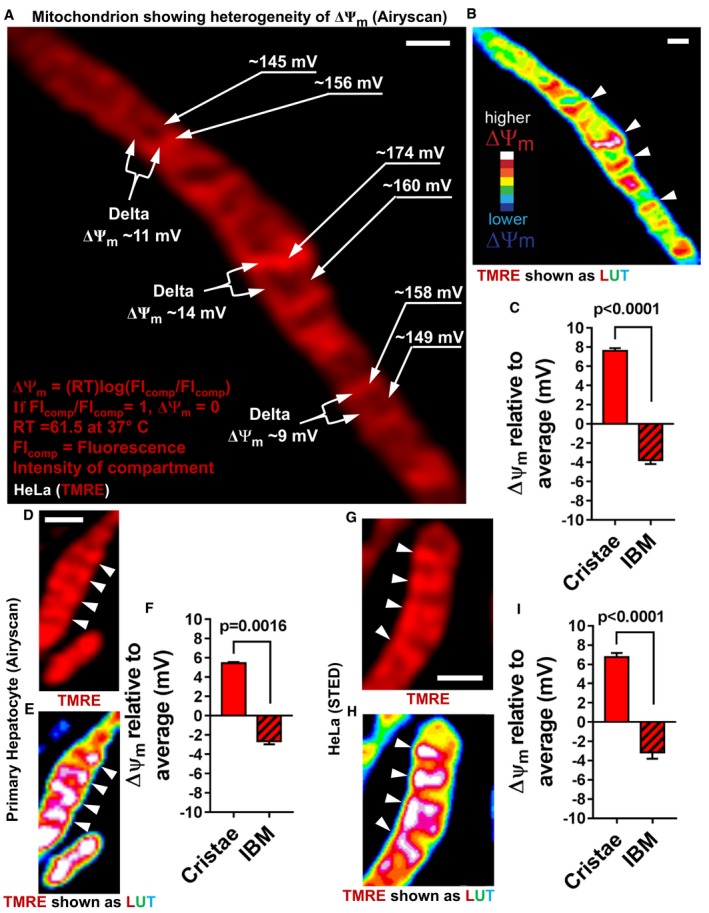
Cristae and IBM have different ΔΨ_m_ ALive‐cell Airyscan image of mitochondrion in HeLa cell, showing different membrane potentials in different mitochondrial regions. Membrane potentials were calculated based on TMRE FI differences between compartments (FI_comp_). Regions of interest from cristae and IBM used for ΔΨ_m_ calculations are labeled with arrows on the right‐hand side of the mitochondrion, using the cytosol as the reference compartment. Labeled with forks on the left‐hand side of the mitochondrion are the calculations of ΔΨ_m_ between individual cristae and their neighboring IBM. Nernst equation used to calculate different voltages. Scale bar = 500 nm.BLUT of mitochondrion in HeLa cell shown in (A), color coding of TMRE FIs on scale of white (most intense) to blue (least intense): LUT scale shown in lower left‐hand corner. Arrowheads indicate cristae. Scale bar = 500 nm. Note that, most‐intense pixels (white) only associate with cristae.CQuantification of ΔΨ_m_ (mV) at cristae and IBM relative to the average ΔΨ_m_ of the whole mitochondrion, calculated as in (A). Note: ΔΨ_m_ at cristae is significantly higher than ΔΨ_m_ at IBM, indicating electrochemical boundaries separate these two regions of the IMM. *N* = 3 independent experiments.D–FLive‐cell Airyscan imaging of mitochondria in cultured primary mouse hepatocytes stained with TMRE. Arrowheads denote cristae. (D) Image showing TMRE‐labeled mitochondria. (E) LUT color coding of TMRE FI. (F) Quantification of ΔΨ_m_ differences using Nernst equation as shown in (A). Scale bar = 500 nm. *N* = 2 independent experiments.G–ILive‐cell STED imaging of mitochondria in HeLa cell stained with TMRM. Arrowheads show cristae. Scale bar = 500 nm. (G) Image showing TMRM‐labeled mitochondrion. (H) LUT color coding of TMRM FI. (I) Quantification of ΔΨ_m_ differences using Nernst equation as shown in (A). Scale bar = 500 nm. *N* = 4 independent experiments. Note that, calculation of ΔΨ_m_ differences between the compartments based on images captured by STED and by Airyscan was very similar.Data information: Data were analyzed with 2‐tailed Student's *t*‐tests, and *P* values < 0.05 were considered statistically significant. Specific *P* values are indicated in the figure. Error bars indicate SEM.

The Nernst equation can be used to quantify ΔΨ_m_ by acquiring the FI of ΔΨ_m_‐sensitive probes (e.g., TMRE). The FIs of the probes at different subcellular compartments can be used to extrapolate the differences in concentrations of the probe, which are needed to calculate the difference in ΔΨ_m_ between compartments (Ehrenberg *et al*, 1988; Farkas *et al*, 1989; Loew *et al*, 1993; Wikstrom *et al*, 2007; Twig *et al*, 2008). We used the average TMRE FI of the mitochondria as a reference point to calculate the ΔΨ_m_ of the different compartments, an approach similar to that employed in multi‐electrode ECG. We found the voltage at cristae to be significantly higher than at IBM (Fig 4A and C). These data indicate that the hetero‐potential along the IMM consists of at least two basic segments—the cristae and the IBM. Representing pixel intensities in pseudo‐color as a LookUp Table (LUT), where white and blue correspond to the highest and lowest ΔΨ_m_, respectively, it is apparent that the voltage associated with cristae (arrowheads) is generally higher than IBM, emphasizing the electrochemical discontinuity between these contiguous regions of the IMM (Fig 4B).

We next explored whether the ΔΨ_m_ of primary cells would follow the same pattern. We stained mitochondria in primary hepatocytes with TMRE and found ΔΨ_m_ heterogeneity similar to that in HeLa cells, indicating that discrete electrochemical domains also exist along the IMM of differentiated cells with strong mitochondrial oxidative function (Fig 4D–F).

To further confirm the ΔΨ_m_ differences between cristae and IBM observed using Airyscan imaging, we determined whether the ΔΨ_m_ would display the same heterogeneity by super‐resolution microscopy (e.g., STED). Using living HeLa cells stained with TMRM, we found a nearly identical pattern in the heterogeneity of ΔΨ_m_, where, notably, cristae ΔΨ_m_ significantly exceeds that of IBM (Fig 4G–I). Altogether, these data demonstrate that the voltage associated with cristae is significantly higher than that of IBM, which is consistent with the higher concentration of ETC components associated with cristae membranes.

### ΔΨ_m_ differences between cristae and IBM (ΔΨ_Cr‐IBM_) are sensitive to inhibition of *F*_1_ *F*_0_ ATP Synthase and to uncoupling

**Figure 5:**
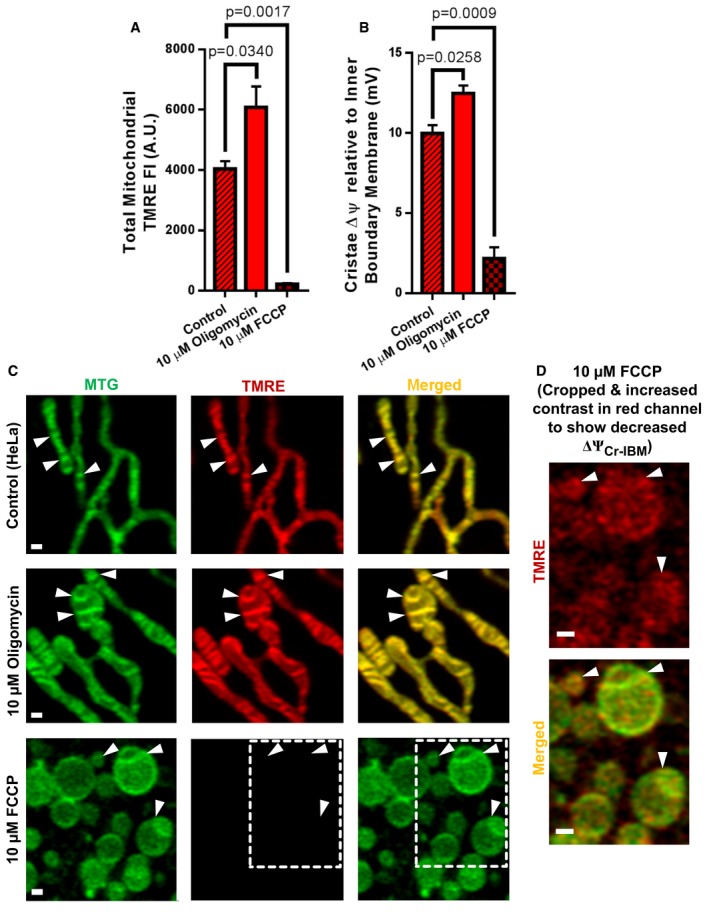
ΔΨ_m_ differences between cristae and IBM are sensitive to *F*_1_ *F*_0_ ATP Synthase inhibition and uncoupling Response of ΔΨ_m_ to the *F*_1_ *F*_0_ ATP Synthase inhibitor, oligomycin (10 μM), compared to the uncoupler, FCCP (10 μM), determined using live‐cell Airyscan imaging of HeLa cells co‐stained with MTG and TMRE.
Quantification of total mitochondrial TMRE FI. *N* = 3 independent experiments.Quantification of cristae ΔΨ_m_ relative to IBM (ΔΨ_Cr‐IBM_) in response to oligomycin compared to FCCP. *F*_1_ *F*_0_ ATP Synthase is exclusively localized to the cristae and significant increase in ΔΨ_Cr‐IBM_ in response to blocking of *F*_1_ *F*_0_ ATP Synthase with oligomycin indicates that differences in TMRE FI between cristae and IBM are driven by oxidative phosphorylation. *N* = 3 independent experiments.Representative Airyscan images showing mitochondria from living HeLa cells stained with MTG and TMRE. Cells were first stained for 1 h and only then treated with oligomycin or FCCP. Images show hyperpolarization of cristae under oligomycin (middle row; arrowheads) and depolarization of cristae under FCCP (bottom row; arrowheads) compared to control (top row; arrowheads). Scale bar = 500 nm. *N* = 3 independent experiments. Note that, the ΔΨ_m_‐independent fraction of TMRE staining is < 5% of the signal in control conditions.Zoomed‐in region of FCCP‐treated mitochondria in (C). Increased contrast was used to visualize TMRE to compensate for TMRE loss induced by FCCP. These images show diminished heterogeneity of ΔΨ_Cr‐IBM_ values (arrowheads). Scale bar = 500 nm.Data information: Data were analyzed with 2‐tailed Student's *t*‐tests, and *P* values < 0.05 were considered statistically significant. Specific *P* values are indicated in the figure. Error bars indicate SEM.

**Figure EV3:**
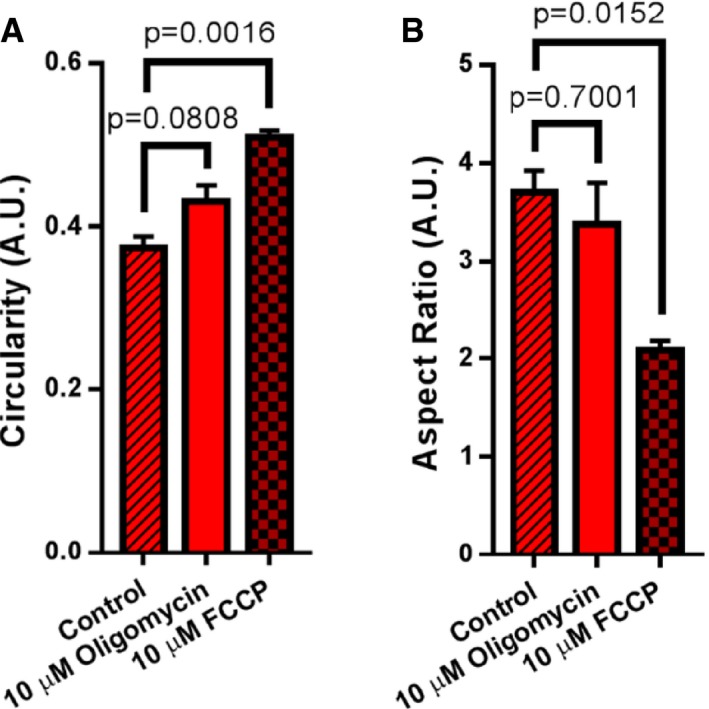
Effects of FCCP and oligomycin on mitochondrial morphology Quantification of mitochondrial circularity, where mitochondria with circularity closer to 1 resemble a circular object and mitochondria with circularity closer to 0 resemble a branched object. Note: FCCP results in significant increase in mitochondrial circularity, consistent with numerous studies indicating FCCP induces fragmentation. *N* = 3 independent experiments.Quantification of mitochondrial aspect ratio (a ratio of the long axis of the mitochondrion to the short axis), where mitochondria with an aspect ratio closer to 1 are rounder, whereas mitochondria higher and higher than 1 are increasingly elongated. Note: FCCP also leads to a significant decrease in aspect ratio, indicating loss of membrane potential results in fragmentation. *N* = 3 independent experiments.Data information: Data were analyzed with 2‐tailed Student's *t*‐tests, and *P* values < 0.05 were considered statistically significant. Specific *P* values are indicated in the figure. Error bars indicate SEM.

*F*_1_ *F*_0_ ATP Synthase is primarily localized to the rims of cristae (Dudkina *et al*, 2005; Strauss *et al*, 2008; Davies *et al*, 2011). If cristae maintain ΔΨ_m_ that is distinct from the IBM, then inhibiting the consumption of the proton gradient by *F*_1_ *F*_0_ ATP Synthase would further increase the difference between IBM and cristae. To assess this hypothesis, we inhibited *F*_1_ *F*_0_ ATP Synthase with oligomycin and measured the difference in the ΔΨ_m_ between the cristae and IBM (ΔΨ_Cr‐IBM_). We found that not only did oligomycin increase the total ΔΨ_m_ (Fig 5A and C), as expected (Farkas *et al*, 1989), but it also increased the ΔΨ_Cr‐IBM_, hyperpolarizing the cristae (Fig 5B and C). Conversely, we tested whether treatment with FCCP, a protonophore that shuttles protons across the IMM, would diminish the ΔΨ_Cr‐IBM_. Consistent with previous studies (Farkas *et al*, 1989; Loew *et al*, 1993), we determined that FCCP decreased the total ΔΨ_m_ (Fig 5A and C) and resulted in mitochondrial fragmentation (Fig EV3A and B) (Duvezin‐Caubet *et al*, 2006; Ishihara *et al*, 2006; Griparic *et al*, 2007). Furthermore, we observed that treatment with FCCP decreased the ΔΨ_Cr‐IBM_ (Fig 5B–D).

### The crista‐junction (CJ) modulators, MICOS complex and Opa1, regulate ΔΨ_Cr‐IBM_

To determine the role of CJs and cristae architecture in the generation of ΔΨ_Cr‐IBM_, we used cellular models in which proteins controlling the formation of cristae and CJs are perturbed. To date, the most notable of these are the family of MICOS‐complex proteins (Rabl *et al*, 2009; Harner *et al*, 2011; Hoppins *et al*, 2011; von der Malsburg *et al*, 2011; Zerbes *et al*, 2012; Barbot *et al*, 2015; Friedman *et al*, 2015; Guarani *et al*, 2015; Barrera *et al*, 2016; Hessenberger *et al*, 2017; Rampelt *et al*, 2017; Wollweber *et al*, 2017) and the GTPase, Opa1 (Frezza *et al*, 2006; Cogliati *et al*, 2016). To determine whether the MICOS complex and/or Opa1 are required to preserve the ΔΨ_Cr‐IBM_, we examined ΔΨ_Cr‐IBM_ in cells with either Opa1 or MICOS‐complex subunits deleted.

**Figure 6:**
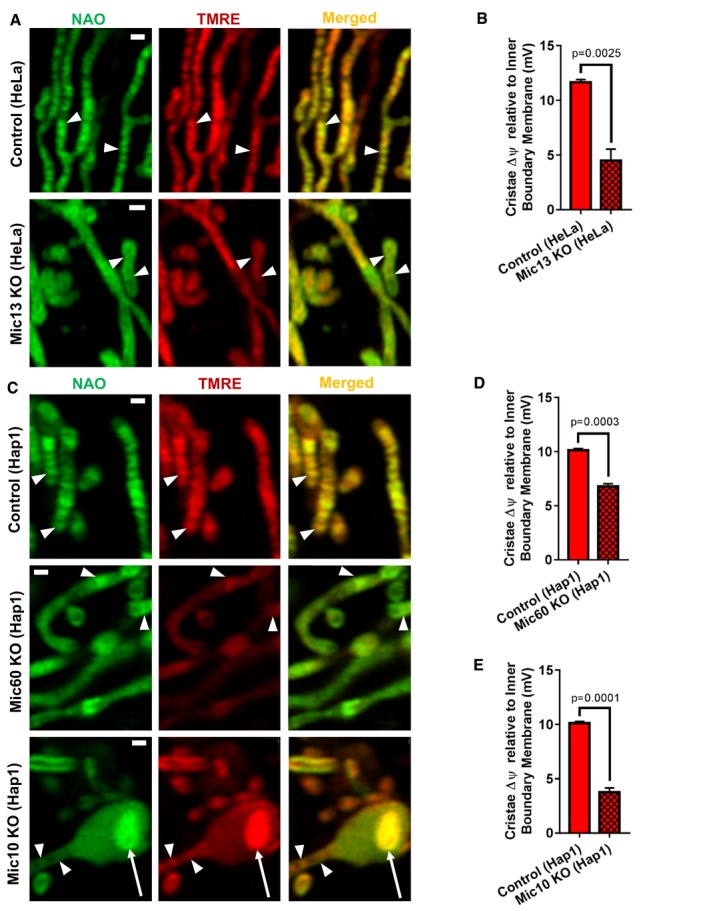
Crista junctions (CJs) regulate the difference in ΔΨ_m_ between cristae and IBM The role of cristae structure as well as CJ formation and sealing on the generation of the difference in ΔΨ_m_ between cristae and IBM were studied by disrupting cristae using Mic10‐, Mic13‐, and Mic60‐deficient cells. Mic13 and Mic60 support cristae formation. Mic10 is essential for CJ formation, and, in its absence, cristae tend to remain as vesicles detached from IBM. Live‐cell Airyscan imaging of TMRE was used in all figure panels and models.
Representative images of mitochondria in control (top row) vs. Mic13‐KO (bottom row) HeLa cells. Note that, TMRE FI in Mic13‐KO mitochondria is distributed more evenly along the IMM, so that ΔΨ_Cr‐IBM_ is decreased (arrowheads). Scale bar = 500 nm. *N* = 3 independent experiments.Quantification of (A), showing deletion of Mic13 in HeLa cells results in significant decrease in ΔΨ_Cr‐IBM_. While Mic13‐KO cells show a substantial decrease in cristae number, the loss of cristae does not appear absolute, making it feasible to compare TMRE FI at cristae relative to IBM. *N* = 3 independent experiments.Representative images of Hap1 control (top row) vs. Mic60 KO (middle row) and Mic10 KO (bottom row); Mic60‐KO mitochondria show very few cristae structures. TMRE staining along the IMM is relatively homogeneous, indicating decreased ΔΨ_Cr‐IBM_. In general, Mic10 KO in Hap1 cells results in decreased TMRE signal intensity at cristae relative to IBM (arrowheads) as compared to control cells. Deletion of Mic10 induces detachment of cristae from IBM (arrows; see Fig 8). Except for the detached cristae vesicles, the TMRE staining is homogeneously distributed in Mic10‐KO cells, indicating decreased ΔΨ_Cr‐IBM_. Scale bar = 500 nm. *N* = 3 independent experiments.Quantification of (C), showing Mic60 KO in Hap1 cells results in significant decrease in ΔΨ_Cr‐IBM_. While Mic60‐KO cells show a marked loss of normal cristae structures, depletion of cristae does not appear absolute, making it possible to compare TMRE FI at cristae relative to IBM. *N* = 3 independent experiments.Quantification of (C), showing Mic10 KO in Hap1 cells results in a significant decrease in ΔΨ_Cr‐IBM_. Note: quantification of ΔΨ_m_ at cristae relative to IBM was performed only on cristae structures that appeared to maintain attachment to IBM. *N* = 3 independent experiments.Data information: Data were analyzed with 2‐tailed Student's *t*‐tests, and *P* values < 0.05 were considered statistically significant. Specific *P* values are indicated in the figure. Error bars indicate SEM.

Firstly, we examined the effect of deleting Mic13 on ΔΨ_Cr‐IBM_ in HeLa cells. While Mic13‐KO cells showed a marked decrease in the number of cristae, we identified some regions of IMM that maintained cristae structures (Fig 6A; arrowheads). Mic13‐KO mitochondria showed decreased heterogeneity of TMRE staining along the IMM and ΔΨ_Cr‐IBM_ was significantly diminished, compared to control HeLa cells (Fig 6A and B). To further address the role of the MICOS complex in regulating the mitochondrial hetero‐potential, we examined cells in which Mic60 or Mic10 were deleted (Fig EV1C). In these cells, we observed a significant diminishment of heterogeneity of TMRE FI along the IMM (Fig 6C), as well as a drop in ΔΨ_Cr‐IBM_ (Fig 6D and E).

**Figure 7:**
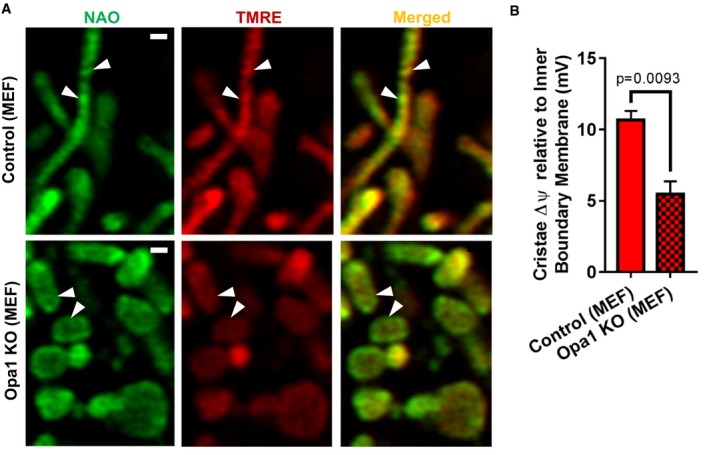
Opa1 regulates the difference in ΔΨ_m_ between cristae and IBM Studies show that Opa1 interacts with MICOS complex, promoting closure of CJs. Thus, we tested whether Opa1 was required to maintain the difference in ΔΨ_m_ between cristae and IBM.
Live‐cell Airyscan images of MEF control (top row) vs. Opa1 KO (bottom row), co‐stained with NAO and TMRE. Arrowheads indicate decreased intensity of TMRE FI at cristae compared to IBM. Note the more even distribution of TMRE staining, indicating the cristae and IBM are relatively equipotential. Scale bar = 500 nm.Quantification of (A), showing Opa1‐KO MEFs have significantly decreased ΔΨ_Cr‐IBM_. *N* = 3 independent experiments.Data information: Data were analyzed with 2‐tailed Student's *t*‐tests, and *P* values < 0.05 were considered statistically significant. Specific *P* values are indicated in the figure. Error bars indicate SEM.

We next studied the effects of Opa1 deletion on ΔΨ_Cr‐IBM_. Various studies have demonstrated that not only does Opa1 associate with components of the MICOS complex (Barrera *et al*, 2016; Glytsou *et al*, 2016), but it appears to have an independent function as a molecular staple, holding the cristae in a closed configuration (Frezza *et al*, 2006). We hypothesized, therefore, that deletion of Opa1 (Fig EV1D), like deletion of components of the MICOS complex, would disrupt the electrochemical boundaries between the cristae and the IBM, thereby equilibrating the potential along the IMM. Our data indicate that deletion of Opa1 indeed results in a significant loss in ΔΨ_Cr‐IBM_ (Fig 7A and B). Altogether, these data indicate that cristae architecture and CJs are essential for the formation of the electrochemical boundaries that allow for ΔΨ_m_ of cristae to remain different from that of IBM.

### The largest differences in ΔΨ_Cr‐IBM_ arise in cristae vesicles, generated by deleting CJ regulators

**Figure 8:**
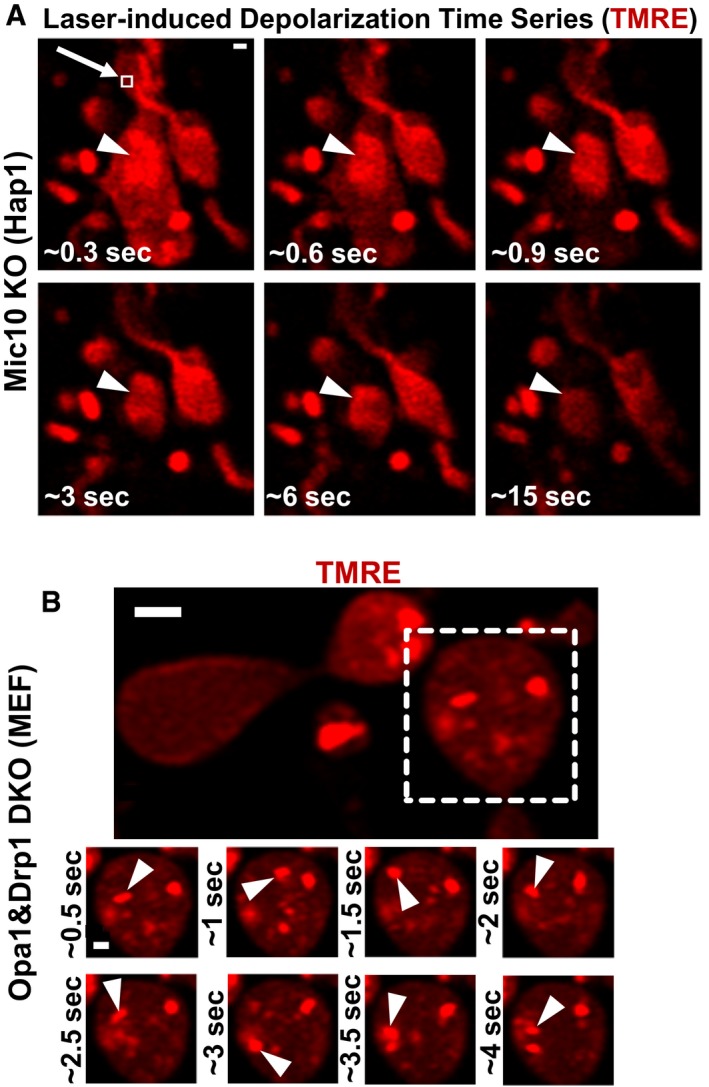
The largest differences in ΔΨ_Cr‐_ _IBM_ arise in cristae vesicles generated by deleting CJ regulators Live‐cell Airyscan images of laser‐induced depolarization time series of Mic10 KO Hap1 cell stained with TMRE. Mitochondrion exposed to rapid, high 2‐photon laser power in region of white box, arrow (˜ 0.3 s); large, hyperpolarized vesicle (arrowhead) remains polarized after the rest of the mitochondrion loses ΔΨ_m_, indicating electrochemical discontinuity between hyperpolarized vesicles and the rest of the mitochondrion, including the IBM. Scale bar = 500 nm. *N* = 2 independent experiments.Live‐cell Airyscan image of time series showing hyperpolarized vesicles (arrowheads; region cropped from dashed white box) inside mitochondria lacking both Opa1 and Drp1. Scale bar = 500 nm. *N* = 2 independent experiments. Free movement of hyperpolarized vesicles within the matrix indicate their detachment from the IBM, representing an extreme case of cristae independency within one mitochondrion (see Movie EV1 for time series).Data information: For quantification of hyperpolarized vesicles, see values in main text.

Intriguingly, in ~ 25% of Mic10 KO cells, we observed structures that appeared to have detached from the IBM and become hyperpolarized (Fig 6C, lowest row, arrow). To test whether these hyperpolarized structures in Mic10 KO cells had no membrane continuity with the IMM, we performed laser‐induced depolarization of mitochondria that included such vesicles. If these hyperpolarized vesicles were unanchored from the IMM, we would expect that they would remain polarized despite the collapse of ΔΨ_m_ elsewhere in the mitochondrion. Figure 8A shows that such a hyperpolarized structure (arrowhead) maintained its ΔΨ_vesicle_ for at least 15 s after the rest of the mitochondrion had depolarized. We determined that the ΔΨ_vesicle‐IBM_ of such vesicles was significantly higher than cristae that maintained attachment to the IBM (ΔΨ_Cr‐IBM_ for Control cristae = 10.11 mV vs. ΔΨ_vesicle‐IBM_ for Mic10 KO vesicles = 25.85 mV; *P* = 0.0005; *N* = 3 independent experiments).

We further hypothesized that deletion of both Opa1 and Drp1 (Fig EV1D) would result in an extreme case of electrochemical discontinuity of the IMM. Indeed, our imaging of Opa1&Drp1 DKO MEFs demonstrates the formation of strongly hyperpolarized vesicles moving haphazardly within the matrix (Fig 8B; Movie EV1 shows time series of hyperpolarized vesicles). Compared to control MEFs that had a ΔΨ_Cr‐IBM_ of 10.67 mV, the hyperpolarized vesicles in the Opa1&Drp1 DKO MEFs showed a ΔΨ_vesicle‐IBM_ of 39.55 mV (*P* = 0.0014; *N* = 3 independent experiments).

Hyperpolarized vesicles were quite rare in control cells, suggesting that they are formed in response to bioenergetic dysfunction and/or impaired cristae formation.

### Different cristae within a mitochondrion can have different ΔΨ_m_, indicating that cristae function as independent bioenergetic units

**Figure 9:**
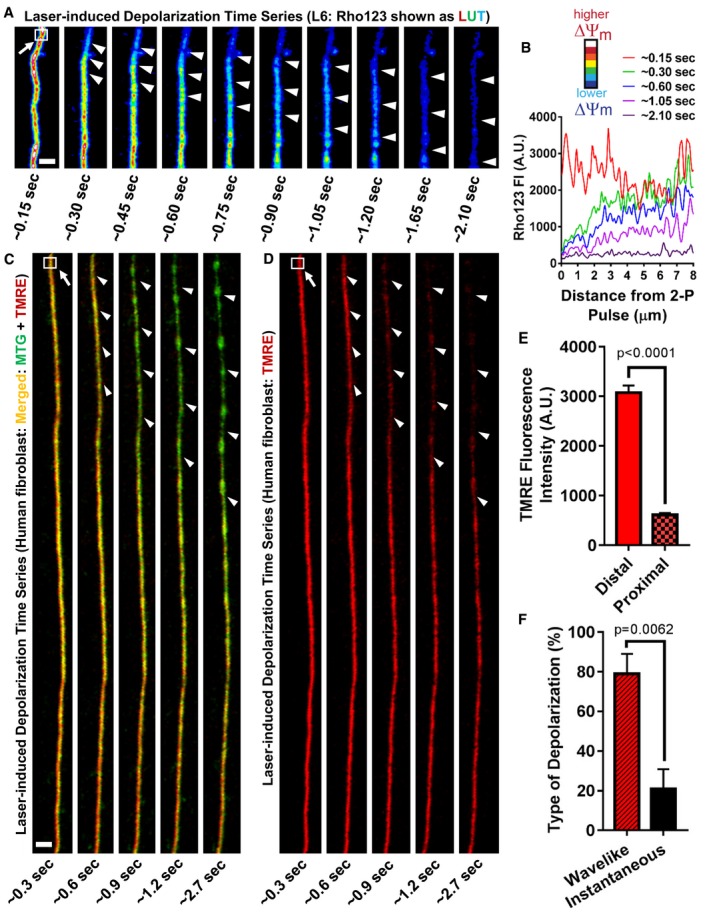
Laser‐induced depolarization results in non‐instantaneous loss of ΔΨ_m_ along the single mitochondrion Laser‐induced mitochondrial membrane depolarization in L6 myoblasts shows gradual depolarization of elements along the IMM, visualized using live‐cell Airyscan microscopy.
ARepresentative images of laser‐induced depolarization time series from L6 myoblast, stained with ΔΨ_m_‐dependent dye, Rho123. Images show LUT color‐coded for Rho123 FI. White and blue pixels represent most‐ and least‐intense ΔΨ_m_, respectively. See legend of LUT colors on the right. Note that, at the first time point (˜ 0.15 s), white arrow points to box marking area of mitochondrion exposed to phototoxic pulse of 2‐photon laser (≤ 5 ms), inducing depolarization. Ensuing frames show wavelike depolarization away from site of perturbation (arrowheads). Scale bar = 500 nm. *N* = 3 independent experiments. (See Movie EV2 for time series.)BProfile plot showing Rho123 pixel intensity as function of distance along mitochondrion in (A). Note that, zero value in the *x*‐axis corresponds to top area of the mitochondrion shown in (A), near site of 2‐photon laser exposure (box). Red line corresponds to frame immediately before depolarization (˜ 0.15 s); green, blue, purple, and dark purple lines correspond to ˜ 0.30, ˜ 0.60, ˜ 1.05, and ˜ 2.10 s, respectively, after initial depolarization, showing wavelike dissipation of ΔΨ_m_ over time.C–FLaser‐induced mitochondrial membrane depolarization in living human skin fibroblasts co‐stained with TMRE and MTG and imaged with Airyscan, showing gradual depolarization of elements along the IMM. (C) Time‐lapse images of merged green (MTG) and red (TMRE) channels following laser‐induced mitochondrial membrane depolarization. Note that, at the first time point (0.3 s), white arrow points to box marking area of mitochondrion exposed to phototoxic pulse of 2‐photon laser (≤ 5 ms), inducing depolarization. Depolarization is marked by the dissipation of the ΔΨ_m_‐dependent dye (TMRE) while the green (MTG) signal persists (arrowheads). Scale bar = 1 μm. *N* = 4 independent experiments. (D) TMRE channel from (C), showing gradual loss of TMRE along the IMM (arrowheads). Note that, although depolarization has largely completed at the area near the phototoxic stimulus already at 0.9 s, some specific regions at the very top maintain ΔΨ_m_ while others depolarize. (E) Quantification of ΔΨ_m_ using TMRE FI from (C, D). Measurement of TMRE pixel intensities immediately after laser‐induced depolarization at site distal (≥ 10 μm) vs. proximal (≤ 1 μm) to box. *N* = 4 independent experiments; see specific *P* values in panel. (F) Quantification of (C, D). Percentage of mitochondria that depolarize in a wavelike (i.e., gradual) vs. instantaneous manner after laser‐induced depolarization. Note: imaging at high temporal resolution (˜ 100–500 ms/frame) reveals wavelike depolarizations predominate, suggesting the ΔΨ_m_ is composed of multiple, disparate electrochemical domains along the IMM. The time scale of propagation of depolarization is slower than the propagation of electrical phenomena. *N* = 4 independent experiments.Data information: Data were analyzed with 2‐tailed Student's *t*‐tests, and *P* values < 0.05 were considered statistically significant. Specific *P* values are indicated in the figure. Error bars indicate SEM.

Previous studies suggest that a single mitochondrion constitutes a single bioenergetic unit (Amchenkova *et al*, 1988; Skulachev, 2001; Glancy *et al*, 2015). A typical experiment would involve laser‐induced depolarization at one tip of a mitochondrion, which would appear to instantaneously result in a complete collapse of the ΔΨ_m_, leading to the conclusion that the entire organelle functions as one electrochemical unit. These time‐lapse experiments determined ΔΨ_m_ at intervals of 5 s or longer after laser‐induced depolarization and were employing microscopes that allowed for single mitochondrion, rather single crista, resolution (Amchenkova *et al*, 1988; Skulachev, 2001). A 5‐s interval, however, is enough time to generate and propagate a soluble signaling molecule and/or changes in membrane architecture that could induce depolarization of independent cristae structures in an entire mitochondrion. Thus, to test whether laser‐induced depolarization reflected (i) an instantaneous collapse in voltage due to the mitochondrion functioning as an uninterrupted electrochemical conduit; or (ii) a gradual loss of voltage due to a chemical signal and/or structural changes propagating through the organelle, we re‐ran the same experiments as previously described but acquired images ~ 20× more rapidly. Visualizing mitochondria from rat myoblasts (L6) using Rho123 (Fig 9A; Movie EV2 shows time series), we exposed a small mitochondrial region (~ 0.5–1 μm^2^) to a rapid, high‐power pulse of the 2‐photon (2‐P) laser (arrow, white box). We then observed mitochondria depolarizing in a wavelike (i.e., gradual) manner: The ΔΨ_m_‐dependent dye dispersed more rapidly near the original site of laser stress, prior to dispersing along areas farther away (arrowheads follow the loss of ΔΨ_m_ over time). The Rho123 signal is displayed as a multicolored LUT, where pixel intensity is color‐coded on a scale where the most‐intense pixels appear white (highest ΔΨ_m_) and the least‐intense pixels appear blue (lowest ΔΨ_m_). Examining the images immediately following laser‐induced depolarization (~ 0.30, ~ 0.45, and ~ 0.60 s), we found that the mitochondrion loses ΔΨ_m_ first in proximity to the site of laser stress, because the pixels become more green and blue, whereas the distal site remains polarized, as indicated by the white and red pixels. In Fig 9B, we plotted the pixel intensity of the mitochondrion in 9A as a function of distance from the top to the bottom at different time points. In the image immediately preceding laser‐induced depolarization (red line), the peaks and valleys occupy a similar range of intensities from one end of the mitochondrion to the other. Following depolarization induced by the 2‐P laser pulse, Rho123 intensity proximal to the site of laser stress drops substantially, while the signal intensity at the distal end remains almost unchanged (green line). These asymmetrical changes in FI are visible at later time points (blue and purple lines), until ~ 2.1 s, when the mitochondrion has lost virtually all of its ΔΨ_m_ (dark purple line).

In previous studies, laser‐induced depolarization experiments were often performed on mitochondria in fibroblasts, which tend to be remarkably elongated. Therefore, fibroblasts represent an optimal system to test the model of the mitochondrion as an “electrical wire” or “power cable”, where loss of ΔΨ_m_ in one place would lead to simultaneous depolarization across the whole organelle (Amchenkova *et al*, 1988; Skulachev, 2001). Staining mitochondria in human fibroblasts with MTG and TMRE, we performed laser‐induced depolarization experiments (Fig 9C). Loading of MTG depends on ΔΨ_m_, but it is retained in mitochondria even after depolarization, because, once inside the organelle, it binds covalently to thiol moieties of various proteins (Presley *et al*, 2003). Partitioning of TMRE to mitochondria, on the other hand, depends entirely on ΔΨ_m_. Following 2‐P laser pulsation (arrow, white box), there is greater and greater loss of TMRE over time, while the signal from MTG remains. Figure 9D, which shows the TMRE channel alone, demonstrates that, even though the mitochondrion begins to depolarize proximal to the site of the initial laser stress, it maintains its ΔΨ_m_, distally, for a period of time. To quantify this asymmetry in ΔΨ_m_, we measured TMRE FI immediately after 2‐P laser pulsation at sites distal to the initial perturbation (≥ 10 μm from white box) vs. proximal (≤ 1 μm from white box; Fig 9E).

Quantification shows that in the seconds following laser‐induced depolarization, mitochondria tend to remain significantly more polarized at sites distant from targeted laser stress, compared to sites close to it. The time gap of seconds for the propagation of the depolarization wave does not support the notion that the IMM functions as a single electrochemical conduit. We next determined the frequency that mitochondria appeared to depolarize in a wavelike vs. instantaneous manner. Time‐lapse imaging at approximately 300 ms/frame, we were significantly more likely to observe wavelike instead of instantaneous depolarization (Fig 9F).

Together, using different cell lines and mitochondrial dyes, our data suggest that the ΔΨ_m_ is organized into multiple, disparate electrochemical domains along the length of the IMM. It further suggests that the laser‐induced depolarization phenomenon propagates within the mitochondrion through the diffusion of a signal and/or structural changes rather than by the membrane acting as a continuous electrochemical element.

**Figure 10:**
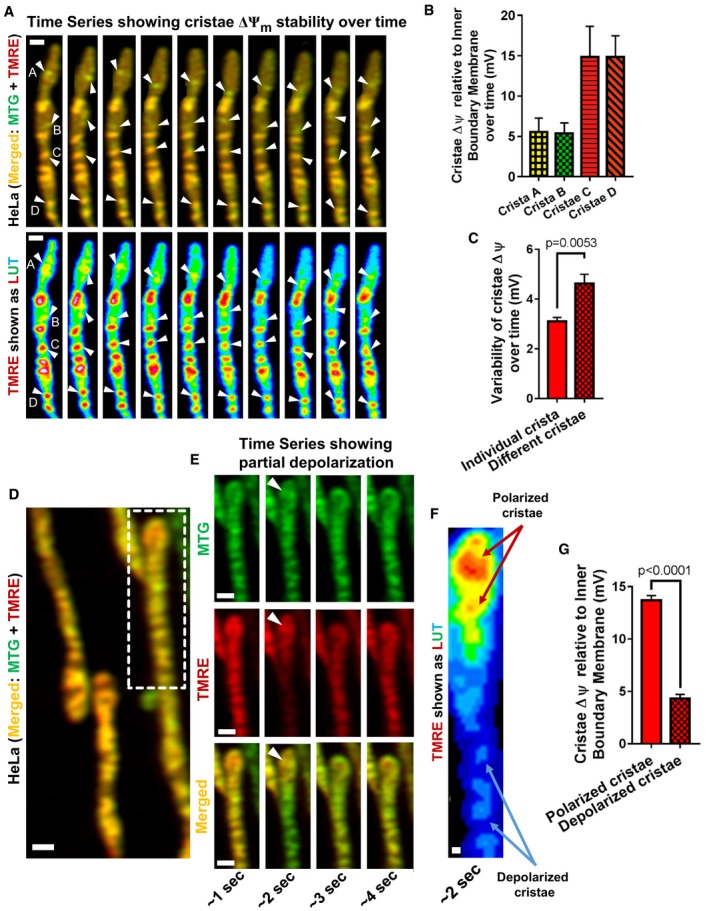
Cristae are unique electrochemical domains, thus constituting independent bioenergetic units We calculated the ΔΨ_Cr‐IBM_ between neighboring cristae over time as a surrogate measure of cristae individuality and functional independence, using live‐cell Airyscan imaging.
AStability of cristae ΔΨ_Cr‐IBM_ over time. Representative images from time series of HeLa cells co‐stained with MTG and TMRE. Top sequence shows merged green (MTG) and red (TMRE) channels. Bottom sequence shows color‐coded LUT of TMRE signal of the same sequence shown above. White arrowheads in the image point to specific cristae that were measured over time. Scale bar = 500 nm. *N* = 5 independent experiments.BQuantification of (A), showing average ΔΨ_Cr‐IBM_ of Cristae “A–D” over time. Note that, Cristae (C) and (D) maintain more than double the ΔΨ_Cr‐IBM_ of Cristae (A) and (B). Error bars indicate STDEV.CQuantification of (A), showing standard deviation in ΔΨ_Cr‐IBM_ (mV) of individual cristae over time compared to the standard deviation in ΔΨ_Cr‐IBM_ (mV) among different cristae along a single mitochondrion. *N* = 5 independent experiments. Note that, there is less variability in ΔΨ_Cr‐IBM_ of a single crista over time within the same mitochondrion compared to the variability of ΔΨ_Cr‐IBM_ among cristae over time, indicating cristae possess unique bioenergetic properties.D–GAiryscan time series of TMRE flickering in HeLa cells co‐stained with MTG and TMRE, showing that depolarization is uncoordinated along the IMM with some cristae depolarizing while neighboring cristae remain polarized. (D) Representative live‐cell Airyscan image from time series showing merged green (MTG) and red (TMRE) channels. Area marked by dashed box is shown in the time series in (E). Scale bar = 500 nm. (E) Time series of cropped region from (D), showing tip of mitochondrion (arrowhead) maintaining ΔΨ_m_ while neighboring region of same mitochondrion depolarizes. The capacity of some cristae to maintain polarity, while adjacent cristae transiently depolarize, indicates cristae can exhibit a significant amount of electrochemical autonomy. Scale bar = 500 nm. *N* = 3 independent experiments. (See [Link], [Link] for time series). (F) Zoomed‐in LUT of mitochondrion from (E) (˜ 2 s), showing some cristae remaining polarized (red) while adjacent cristae depolarize (blue). (See Movie EV5 for time series). Scale bar = 100 nm. (G) Quantification of (D–F), showing that during a flickering event, some cristae maintain their membrane potentials, while other cristae depolarize, resulting in large differences in ΔΨ_Cr‐IBM_ along the individual mitochondrion. *N* = 3 independent experiments.Data information: Data were analyzed with 2‐tailed Student's *t*‐tests, and *P* values < 0.05 were considered statistically significant. Specific *P* values are indicated in the figure. Error bars indicate SEM, except in panel (B), which show STDEV.

If cristae possess a measure of functional autonomy, then it is possible that different cristae within a single mitochondrion could maintain significantly different membrane potentials (ΔΨ_Cr_) from each other. To determine whether one crista within a single mitochondrion has higher ΔΨ_Cr_ compared to a neighboring crista, we first defined the level of stability of ΔΨ_Cr_ of individual cristae and then asked if the difference between two cristae is larger than the ΔΨ_Cr_ fluctuation observed over time in a single crista. To explore this possibility, we performed time‐lapse imaging of mitochondria stained with MTG and TMRE, and we examined the stability of ΔΨ_Cr_ generated by different cristae over time. The membrane potential of each crista was calculated by referencing it to the IBM to produce a value of ΔΨ_Cr‐IBM_. Figure 10A shows an example of four different cristae—“A–D” (arrowheads)—that we followed for a period of ~ 10 s. Figure 10B shows that the average ΔΨ_Cr‐IBM_ over time of Cristae C and D is more than double that of Cristae A and B, indicating that cristae maintain different membrane potentials. To further examine their relative functional stability over time, we quantified the standard deviation of ΔΨ_Cr‐IBM_ within each crista over time and compared it to the standard deviation of ΔΨ_Cr‐IBM_ between cristae over time. Our data indicate that, over a period of ~ 10 s, there are significantly larger differences between cristae within a single mitochondrion than the variability recorded from any individual crista over time (Fig 10C). To further investigate the extent to which cristae could exhibit functional autonomy, we studied time‐lapse images of mitochondria stained with MTG and TMRE and discovered instances where mitochondria would undergo partial depolarization (Fig 10D–F; arrowhead, arrows; [Link], [Link], [Link] show time series of partial depolarization from E, F). Remarkably, instances of partial depolarization of a single mitochondrion revealed cristae that remained polarized despite the depolarization of adjacent cristae. Analyzing the specific frames showing partial depolarization, we quantified the ΔΨ_Cr‐IBM_ of the polarized cristae vs. depolarized cristae and determined that the polarized cristae maintained a higher ΔΨ_Cr‐IBM_ than neighboring cristae that appeared to have depolarized (Fig 10G). Altogether, our data demonstrate that cristae display a degree of functional autonomy, highlighting a new role of these critical structures as independent bioenergetic units.

## Discussion

ΔΨ_m_ is the main driving force for proton re‐entry through *F*_1_ *F*_0_ ATP Synthase into the mitochondrial matrix (Mitchell, 1961; Mitchell & Moyle, 1969). In this study, we provide evidence, for the first time, that cristae maintain higher ΔΨ_m_ than IBM and that each individual crista within the same mitochondrion can maintain ΔΨ_m_ distinct from neighboring cristae. Using the LSM880 with Airyscan and STED microscopy, we directly visualized the relationship of the ΔΨ_m_ to the IMM in respiring mitochondria. Our observation that cristae and IBM have heterogeneous ΔΨ_m_ is based on differential partitioning of ΔΨ_m_‐dependent probes to the different segments of the IMM. We provide several lines of evidence to support that this differential partitioning of the ΔΨ_m_‐dependent probes is reflecting ΔΨ_m_ and not an artifact of dye binding to membranes: (i) We repeated the observation with the unique ΔΨ_m_‐dependent probes, TMRE, TMRM, and Rho123 (Farkas *et al*, 1989; Loew *et al*, 1993; Duchen, 2004). If partitioning to cristae were based on non‐Nernstian binding, it is unlikely to occur with different dyes. (ii) ΔΨ_Cr‐IBM_ increased in response to oligomycin and decreased in response to FCCP. (iii) Partitioning to the cristae was decreased in models lacking MICOS‐complex subunits or Opa1. (iv) Analyzing flickering events, heterogeneity of TMRE FI along the mitochondrion was markedly decreased during instances of depolarization and reestablished following restoration of ΔΨ_m_. This confirms that TMRE signal stemming from ΔΨ_m_‐independent binding of TMRE to the cristae in our experimental system was negligible. (v) The data relating to ΔΨ_Cr‐IBM_ obtained by Airyscan technology were confirmed by STED microscopy. The imaging was performed in different laboratories with different batches of HeLa cells. (vi) We provided data to confirm the same observations in MEFs, HeLa cells, Hap1 cells, L6 myoblasts, H1975 cells, primary mouse hepatocytes, and human fibroblasts. Altogether, this evidence strongly supports the conclusion that cristae and IBM are sufficiently separated, thus being electrochemically insulated from each other, to allow for different membrane potentials to co‐exist along the IMM. A limitation of this methodology is the lack of dyes that are completely independent of ΔΨ_m_ or cardiolipin content. As a result, the NAO and MTG staining of IBM appeared weaker than that of cristae, precluding ratiometric imaging. This limitation only pertains to the differences in ΔΨ_m_ of cristae vs. IBM but would not affect conclusions related to the ΔΨ_m_ differences between different cristae within the same mitochondrion. Moreover, the six points listed above support that the TMRE FI differences we observed between cristae and IBM indeed reflect ΔΨ_m_ differences.

**Figure 11:**
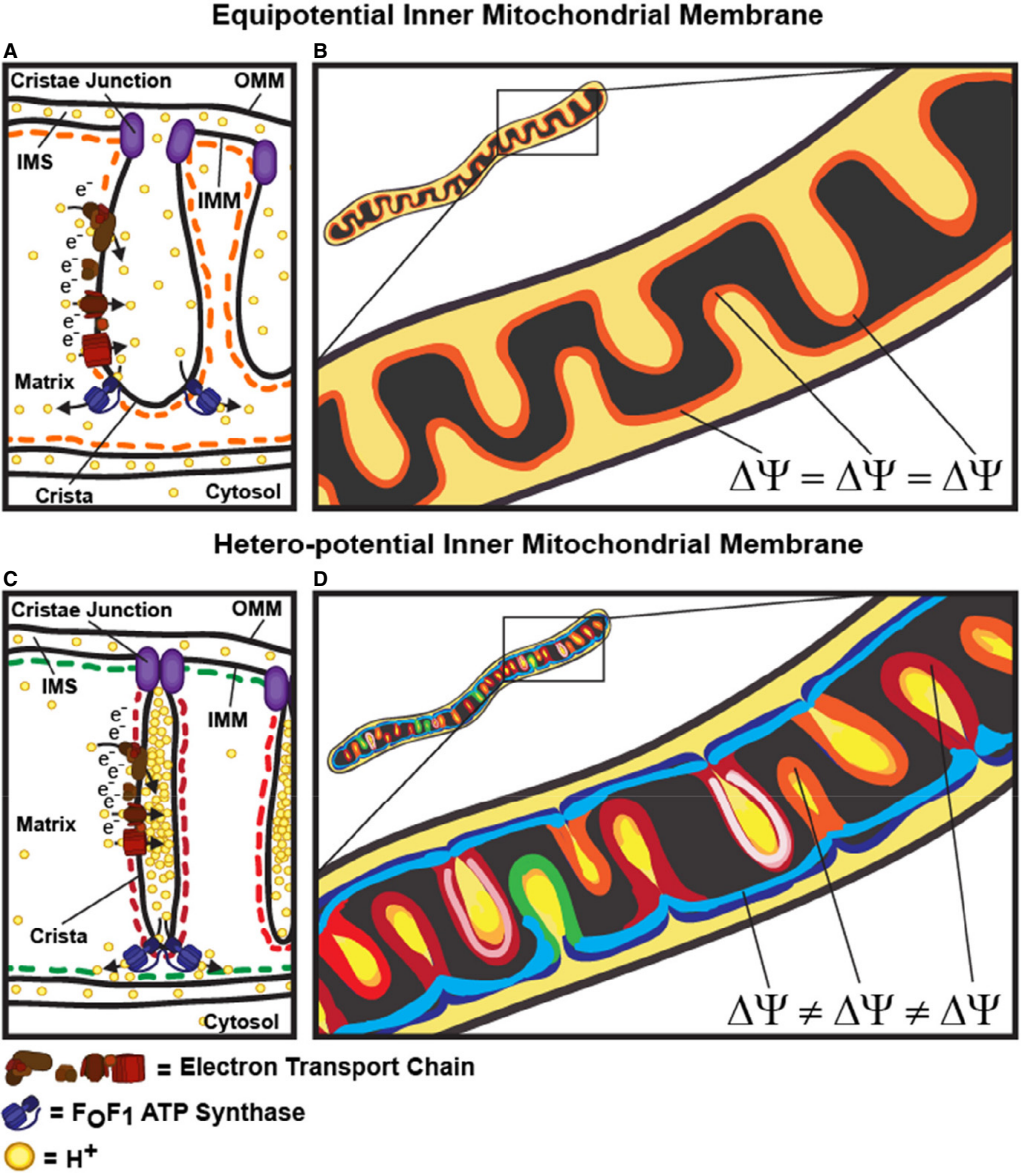
Model of cristae as individual bioenergetic units An equipotential representation of the IMM. In this model, the IMM functions as a continuous electrical cable where cristae and IBM have similar ΔΨ_m_ and where all cristae respond simultaneously to any change in ΔΨ_m_ occurring in the mitochondrion where they reside. Transition from a hetero‐potential to an equipotential, via cleavage of Opa1, for example, could play a role in normal mitochondrial physiology when high conductance and low ATP synthesis are favored, such as thermogenesis or adaptation to excess nutrient.Cartoon showing IMM as equipotential.Model of mitochondrial hetero‐potential, showing cristae as individual bioenergetic units. Electrochemical autonomy of cristae protects the mitochondrion confronting a bioenergetic crisis, where dysfunction of one or more cristae would not inevitably cause the entire organelle to fail. In this model, cristae and IBM can have different membrane potentials. Closed CJs generate significant differences in charge between the cristae membranes and IBM, which could be related to the concentration of ETC subunits in the lateral side of the cristae and *F*_1_ *F*_0_ ATP Synthase on the cristae rims.Cartoon showing IMM as hetero‐potential.

After confirming that the heterogeneous partitioning of ΔΨ_m_‐dependent probes was in fact due to differences in ΔΨ_m_, we examined the possibility that different cristae within the same mitochondrion could function as unique electrochemical domains and thus as independent bioenergetic units. We observed that, when imaging at high temporal resolution, laser‐induced depolarization of elongated mitochondria did not tend to result in a simultaneous collapse of ΔΨ_m_ across the entire organelle but rather in a rapid, wavelike depolarization. This discrepancy from earlier studies can be attributed to the fact that previous assays involving laser‐induced damage did not image the mitochondria rapidly enough after perturbing a small region of the organelle—for example, acquisition of a single frame expended 5–10 s (Amchenkova *et al*, 1988; Skulachev, 2001). Critical delays, either in the time it takes to acquire a single frame of a time series (Amchenkova *et al*, 1988) or in the duration of the perturbation itself (Glancy *et al*, 2015), will increase the likelihood of missing possible spatial differences in depolarization (originating close to the site of laser‐induced damage). Our study suggests that, unless imaging at ≥ 1 frame(s) per second, observing partial depolarization events is improbable. The tendency for one mitochondrion to exhibit localized depolarization indicates that a single organelle does not resemble an electrical wire (Fig 11A and B) but rather appears to function more like a configuration of interconnected batteries (Fig 11C and D).

Our high‐ or super‐resolution imaging of the IMM stained with ΔΨ_m_‐dependent dyes is consistent with a model of cristae as individual, interconnected batteries. The ΔΨ_m_ is not homogeneous along the IMM but shows significant differences between the cristae and IBM, demonstrating electrochemical discontinuity between the otherwise physically connected membranes. The relatively more negative ΔΨ of the cristae membrane is expected to exert a stronger electrical force, keeping protons proximal to the cristae membrane. Indeed, our study can provide an explanation for heterogeneous distribution of protons along the cristae membrane and between the cristae and the IMS (Busch *et al*, 2013; Rieger *et al*, 2014; Pham *et al*, 2016). Following deletions of various proteins involved in regulating cristae architecture, including CJ formation, we observed a decrease in the difference between the ΔΨ_m_ at the cristae membranes relative to the IBM. These data suggest that opening of CJs tends to equalize the ΔΨ_m_ between cristae and IBM by a mechanism that requires further study. The generation of hyperpolarized vesicles, following different genetic perturbations that eliminate CJs, reinforces our general observation that increasing compartmentalization of the IMM, in turn, intensifies the electrochemical gradient. Lastly, our time‐lapse imaging experiments show that the ΔΨ_Cr‐IBM_ of an individual crista is less variable over time compared to the ΔΨ_Cr‐IBM_ among different cristae within the same mitochondrion. Furthermore, we observe that, during transient depolarization, some cristae can maintain polarity despite the collapse in ΔΨ_m_ of adjacent cristae. In summary, our data support a new paradigm where each crista maintains its own ΔΨ_m_ that is different from both the IBM and from neighboring cristae.

This study raises interesting questions as to why mitochondria organize the ΔΨ_m_ in this way. One advantage, for example, could be related to the fact that ΔΨ_m_ constitutes the main energy available to drive protons through *F*_1_ *F*_0_ ATP Synthase to produce ATP. As such, the localization of *F*_1_ *F*_0_ ATP Synthase at the cristae rims appears to be advantageous in terms of proximity to the batteries. Another possible advantage could be compartmentalization of ΔΨ_m_ in each crista may serve as a safeguard mechanism restricting the impact of localized damage. In the case of the equipotential model, where the inner membrane of the entire mitochondrion represents a single capacitor, a breach in membrane integrity in one crista would cause a collapse in voltage in all cristae and compromise the function of the whole organelle. If, on the other hand, the IMM could maintain numerous, discrete electrochemical gradients, like a group of batteries, then failure of one or more would not invariably jeopardize the entire mitochondrion. This may be of particular relevance in cells harboring a highly interconnected mitochondrial network as opposed to cells with less elongated and/or branched mitochondria. Furthermore, the hetero‐potential model suggests that cristae with higher ΔΨ_Cr‐IBM_ could compensate for cristae with impaired function.

Hyperpolarized and depolarized IMM potentials are associated with different states of respiration. While both uncoupling and an increased rate of ATP synthesis dissipate ΔΨ_m_, a decrease in ATP synthesis may result in hyperpolarization and increased ROS production. The hetero‐potential model of the mitochondrion allows for different cristae to serve different functions. In this model, some cristae could be more dedicated to ATP synthesis, whereas neighboring cristae could play a role in ROS signaling. The hetero‐potential model further allows for the consideration that different cristae may engage in primarily complex II vs. complex I respiration, which are associated with different membrane potentials and could be driven by different fuels.

In conclusion, our study identifies a new parameter of mitochondrial function: a mitochondrial hetero‐potential arising from the compartmentalization of the ΔΨ_m_ along the IMM. These findings have wide‐ranging implications for human health, because many diseases and medical complications—for example, dominant optic atrophy (Amati‐Bonneau *et al*, 2008; Zanna *et al*, 2008), ischemia‐reperfusion injury (Birk *et al*, 2013), and even aging (Daum *et al*, 2013)—are associated with severe perturbations in cristae structure. Pathogenic changes to cristae architecture may undermine the autonomy of cristae bioenergetics, leading to the conversion of the IMM into an equipotential membrane. Consequently, damage inflicted to any isolated region may impact the bioenergetics of the entire mitochondrion, rendering the cell more vulnerable to mitochondrial toxicants and metabolic stress. Restoring the architecture of the IMM, therefore, could represent a viable approach to reestablishing the disparate electrochemical gradients that are the basis for normal mitochondrial function. This study emphasizes the need to directly explore the relationship between cristae structure and function to further elucidate the etiology of such diseases and, in turn, foster more effective therapies for treating them.

## Materials and Methods

### Cell culture

H1975 (sh‐Scramble and sh‐PTPMT1) cells were grown in RPMI‐1640 (31800‐022), supplemented with sodium bicarbonate, Pen/Strep, sodium pyruvate, HEPES, and 10% FBS, and cultured in 5% CO_2_. Patient‐derived fibroblasts were grown in DMEM (12100‐046), supplemented with sodium bicarbonate, Pen/Strep, sodium pyruvate, HEPES, and 10% FBS, and cultured in 5% CO_2_. L6, MEF, and HeLa cells were cultured in DMEM (12100‐046), supplemented with sodium bicarbonate, Pen/Strep, sodium pyruvate, HEPES, and 10% FBS, and cultured in 5% CO_2_. Hap1 cells were cultured in Iscove's modified Dulbecco's medium (IMDM) (I3390‐500 ml), supplemented with 20% FBS, Pen/Strep, and 2 mM Glutamax. Primary cultures were isolated from unmodified C57BL6/J males and females, 12–28 weeks old, procured from Jackson laboratories and bred in our facility. Mice were provided with water and food *ad libitum*, and housed 2–5 mice per cage, 12‐h light:dark cycle and at a room temperature of 22–24°C.

### KD and KO models

Human H1975 cells expressing shRNA targeting human PTPMT1 were generated using a pLKO.1 vector harboring shRNA (PTPMT1) Clone ID XM_374879.1‐547s1c1 (Sigma‐Aldrich). Lipofectamine 2000 (Thermo Fisher) with PLKO.1 shRNA (PTPMT1) plasmid, lentiviral packaging plasmids pMDLg/pRRE, pRSV‐Rev, and enveloping plasmid pMD2.G (Addgene #12251, 12253, 12259) were used to transfect 293T cells to generate lentiviral particles. Forty‐eight hours post‐transfection, viral media was removed from 293T cells and centrifuged for 5 min at 300 *g*. Viral supernatant was then passed through 0.45‐μM filter. Target cells were plated at 60% confluency and infected with 1× polybrene and viral supernatant. Viral media was removed and substituted for fresh culture media after 24 h, and incubated for an additional 24 h, followed by puromycin selection. Mic13 KO model was generated through Crispr/Cas9 genome editing (preprint: Kondadi *et al*, 2019), according to previously described methods (Anand *et al*, 2016). Mic10 and Mic60 KO cells were made by Horizon (UK) (preprint: Kondadi *et al*, 2019). Opa1 and Opa1/DRP1 were also generated through Crispr/Cas9 genome editing.

### Live‐cell imaging

Cells were plated in CELLview 4‐compartment glass‐bottom tissue culture dishes (Greiner Bio‐One, 627870), PS, 35/10 mm. Dyes (100 nM 10‐*N*‐nonyl acridine orange, 15 nM TMRE, 5 μM Rho123, and/or 200 nM MitoTracker Green; Invitrogen) were added to cell culture media and incubated 1–3 h prior to live‐cell imaging with the alpha Plan‐Apochromat 100×/1.46 Oil DIC M27 objective on the Zeiss LSM 880 with Airyscan. Prior to image analysis, raw .czi files were automatically processed into deconvoluted Airyscan images using the Zen software. HeLa cells imaged with STED were stained with 50 nM TMRM (Invitrogen) for 30 min before imaging. Leica SP8 LSM, fitted with STED module, was used to perform live‐cell super‐resolution imaging.

### Image analysis

Processed Airyscan images were analyzed using ImageJ (Fiji) software. Briefly, prior to cell cropping and quantification, background was subtracted from all images using a rolling ball filter = 50. After developing analysis protocols, we designed macros for high‐throughput image quantification and analysis. For representative images in figures, we applied the Window/Level function when demonstrating relevant changes in pixel intensities; when specifically comparing cellular structures, we adjusted pixel intensities to optimally demonstrate relevant changes in structure. Images acquired with STED microscopy were deconvoluted using Huygens deconvolution software.

### Immunoblotting

SDS–PAGE assays were conducted by separating 20–30 μg of protein on precast polyacrylamide NuPAGE gels (Thermo Fisher) and transferred to nitrocellulose membranes in transfer buffer containing 10% methanol. Proteins were probed with the following primary and secondary antibodies: Opa1 1:1,000 (BD Biosciences, 612606); HSP60 1:10,000 (Abcam, ab46798); Drp1/DLP1 1:1,000 (BD Biosciences, 611112); PTPMT1 1:1,000 (Sigma‐Aldrich, HPA043932); β‐actin 1:10,000 (Cell Signaling Technology, BH10D10); TOM20 1:2,000 (Abcam, ab78547); Mic10/C1orf151 1:1,000 (MA5‐26031); Mic13/C19orf70 1:1,000 (ProteinTech 25514‐1‐AP); Mic60/mitofilin 1:1,000 (ProteinTech 10179‐1‐AP); anti‐mouse IgG, HRP‐linked antibody (Cell Signaling Technology, 7076S); and anti‐rabbit IgG, HRP‐linked antibody (Cell Signaling Technology, 7074S). Proteins were detected and imaged using SuperSignal West Femto Maximum Sensitivity Substrate (Thermo Fisher) and ChemiDoc MP Imaging System (Bio‐Rad), respectively. Densitometry was performed using ImageJ (Fiji).

### Statistical analysis

All statistical analyses were performed on GraphPad Prism and Microsoft Excel. Data sets were subjected to D'Agostino–Pearson omnibus and/or Shapiro–Wilk normality tests to assess whether data were normally distributed. Data were subjected to 2‐tailed Student's *t*‐tests, and *P* values < 0.05 were considered statistically significant. Error bars represent SEM, unless otherwise indicated. *N* = the number of independent experiments. On average, ~ 20 cells were analyzed per independent experiment per condition. Statistical analysis was performed on the averages from independent experiments.

## Author contributions

DMW and MS designed the study, made the figures, and wrote the article. DMW and MS performed all of the Airyscan imaging, Western blotting, as well as data analysis. STB generated the sh‐Scramble and sh‐PTPMT1 cell lines. AKK performed the STED imaging. RA created the Mic13 KO cell line. ASR, AMB, and DBS provided valuable recommendations, contributing to the study design and reagents. OSS and ML provided supervision, designed the study, and wrote the article.

## Conflict of interest

The authors declare that they have no conflict of interest.

## Supporting information



Expanded View Figures PDF

Movie EV1

Movie EV2

Movie EV3

Movie EV4

Movie EV5

Source Data for Expanded View

Review Process File

## References

[embj2018101056-bib-0001] Amati‐Bonneau P , Valentino ML , Reynier P , Gallardo ME , Bornstein B , Boissiere A , Campos Y , Rivera H , de la Aleja JG , Carroccia R *et al* (2008) OPA1 mutations induce mitochondrial DNA instability and optic atrophy ‘plus’ phenotypes. Brain 131: 338–351 18158317 10.1093/brain/awm298

[embj2018101056-bib-0002] Amchenkova AA , Bakeeva LE , Chentsov YS , Skulachev VP , Zorov DB (1988) Coupling membranes as energy‐transmitting cables. I. Filamentous mitochondria in fibroblasts and mitochondrial clusters in cardiomyocytes. J Cell Biol 107: 481–495 3417757 10.1083/jcb.107.2.481 PMC2115217

[embj2018101056-bib-0003] Anand R , Strecker V , Urbach J , Wittig I , Reichert AS (2016) Mic13 is essential for formation of crista junctions in mammalian cells. PLoS ONE 11: e0160258 27479602 10.1371/journal.pone.0160258 PMC4968808

[embj2018101056-bib-0004] Barbot M , Jans DC , Schulz C , Denkert N , Kroppen B , Hoppert M , Jakobs S , Meinecke M (2015) Mic10 oligomerizes to bend mitochondrial inner membranes at cristae junctions. Cell Metab 21: 756–763 25955211 10.1016/j.cmet.2015.04.006

[embj2018101056-bib-0005] Barrera M , Koob S , Dikov D , Vogel F , Reichert AS (2016) OPA1 functionally interacts with MIC60 but is dispensable for crista junction formation. FEBS Lett 590: 3309–3322 27587279 10.1002/1873-3468.12384

[embj2018101056-bib-0006] Birk AV , Liu S , Soong Y , Mills W , Singh P , Warren JD , Seshan SV , Pardee JD , Szeto HH (2013) The mitochondrial‐targeted compound SS‐31 re‐energizes ischemic mitochondria by interacting with cardiolipin. J Am Soc Nephrol 24: 1250–1261 23813215 10.1681/ASN.2012121216 PMC3736700

[embj2018101056-bib-0007] Busch KB , Deckers‐Hebestreit G , Hanke GT , Mulkidjanian AY (2013) Dynamics of bioenergetic microcompartments. Biol Chem 394: 163–188 23104839 10.1515/hsz-2012-0254

[embj2018101056-bib-0008] Cogliati S , Enriquez JA , Scorrano L (2016) Mitochondrial cristae: where beauty meets functionality. Trends Biochem Sci 41: 261–273 26857402 10.1016/j.tibs.2016.01.001

[embj2018101056-bib-0009] Daum B , Walter A , Horst A , Osiewacz HD , Kuhlbrandt W (2013) Age‐dependent dissociation of ATP synthase dimers and loss of inner‐membrane cristae in mitochondria. Proc Natl Acad Sci USA 110: 15301–15306 24006361 10.1073/pnas.1305462110 PMC3780843

[embj2018101056-bib-0010] Davies KM , Strauss M , Daum B , Kief JH , Osiewacz HD , Rycovska A , Zickermann V , Kuhlbrandt W (2011) Macromolecular organization of ATP synthase and complex I in whole mitochondria. Proc Natl Acad Sci USA 108: 14121–14126 21836051 10.1073/pnas.1103621108 PMC3161574

[embj2018101056-bib-0011] Duchen MR , Leyssens A , Crompton M (1998) Transient mitochondrial depolarizations reflect focal sarcoplasmic reticular calcium release in single rat cardiomyocytes. J Cell Biol 142: 975–988 9722610 10.1083/jcb.142.4.975 PMC2132882

[embj2018101056-bib-0012] Duchen MR (2004) Mitochondria in health and disease: perspectives on a new mitochondrial biology. Mol Aspects Med 25: 365–451 15302203 10.1016/j.mam.2004.03.001

[embj2018101056-bib-0013] Dudkina NV , Heinemeyer J , Keegstra W , Boekema EJ , Braun HP (2005) Structure of dimeric ATP synthase from mitochondria: an angular association of monomers induces the strong curvature of the inner membrane. FEBS Lett 579: 5769–5772 16223490 10.1016/j.febslet.2005.09.065

[embj2018101056-bib-0014] Duvezin‐Caubet S , Jagasia R , Wagener J , Hofmann S , Trifunovic A , Hansson A , Chomyn A , Bauer MF , Attardi G , Larsson NG *et al* (2006) Proteolytic processing of OPA1 links mitochondrial dysfunction to alterations in mitochondrial morphology. J Biol Chem 281: 37972–37979 17003040 10.1074/jbc.M606059200

[embj2018101056-bib-0015] Ehrenberg B , Montana V , Wei M , Wuskell JP , Loew LM (1988) Membrane potential can be determined in individual cells from the Nernstian distribution of cationic dyes. Biophys J 53: 785–794 3390520 10.1016/S0006-3495(88)83158-8 PMC1330255

[embj2018101056-bib-0016] Farkas DL , Wei M , Febbroriello P , Carson JH , Loew LM (1989) Simultaneous imaging of cell and mitochondrial membrane potentials. Biophys J 56: 1053–1069 2611324 10.1016/S0006-3495(89)82754-7 PMC1280610

[embj2018101056-bib-0017] Frezza C , Cipolat S , Martins de Brito O , Micaroni M , Beznoussenko GV , Rudka T , Bartoli D , Polishuck RS , Danial NN , De Strooper B *et al* (2006) OPA1 controls apoptotic cristae remodeling independently from mitochondrial fusion. Cell 126: 177–189 16839885 10.1016/j.cell.2006.06.025

[embj2018101056-bib-0018] Friedman JR , Mourier A , Yamada J , McCaffery JM , Nunnari J (2015) MICOS coordinates with respiratory complexes and lipids to establish mitochondrial inner membrane architecture. Elife 4: e07739 10.7554/eLife.07739 PMC4434539 25918844

[embj2018101056-bib-0019] Glancy B , Hartnell LM , Malide D , Yu ZX , Combs CA , Connelly PS , Subramaniam S , Balaban RS (2015) Mitochondrial reticulum for cellular energy distribution in muscle. Nature 523: 617–620 26223627 10.1038/nature14614 PMC6988728

[embj2018101056-bib-0020] Glytsou C , Calvo E , Cogliati S , Mehrotra A , Anastasia I , Rigoni G , Raimondi A , Shintani N , Loureiro M , Vazquez J *et al* (2016) Optic atrophy 1 is epistatic to the core MICOS component MIC60 in mitochondrial cristae shape control. Cell Rep 17: 3024–3034 27974214 10.1016/j.celrep.2016.11.049 PMC5186903

[embj2018101056-bib-0021] Griparic L , Kanazawa T , van der Bliek AM (2007) Regulation of the mitochondrial dynamin‐like protein Opa1 by proteolytic cleavage. J Cell Biol 178: 757–764 17709430 10.1083/jcb.200704112 PMC2064541

[embj2018101056-bib-0022] Guarani V , McNeill EM , Paulo JA , Huttlin EL , Frohlich F , Gygi SP , Van Vactor D , Harper JW (2015) QIL1 is a novel mitochondrial protein required for MICOS complex stability and cristae morphology. Elife 4: e06265 10.7554/eLife.06265 PMC4439739 25997101

[embj2018101056-bib-0023] Harner M , Korner C , Walther D , Mokranjac D , Kaesmacher J , Welsch U , Griffith J , Mann M , Reggiori F , Neupert W (2011) The mitochondrial contact site complex, a determinant of mitochondrial architecture. EMBO J 30: 4356–4370 22009199 10.1038/emboj.2011.379 PMC3230385

[embj2018101056-bib-0024] Hessenberger M , Zerbes RM , Rampelt H , Kunz S , Xavier AH , Purfurst B , Lilie H , Pfanner N , van der Laan M , Daumke O (2017) Regulated membrane remodeling by Mic60 controls formation of mitochondrial crista junctions. Nat Commun 8: 15258 28561061 10.1038/ncomms15258 PMC5460017

[embj2018101056-bib-0025] Hoppins S , Collins SR , Cassidy‐Stone A , Hummel E , Devay RM , Lackner LL , Westermann B , Schuldiner M , Weissman JS , Nunnari J (2011) A mitochondrial‐focused genetic interaction map reveals a scaffold‐like complex required for inner membrane organization in mitochondria. J Cell Biol 195: 323–340 21987634 10.1083/jcb.201107053 PMC3198156

[embj2018101056-bib-0026] Ishihara N , Fujita Y , Oka T , Mihara K (2006) Regulation of mitochondrial morphology through proteolytic cleavage of OPA1. EMBO J 25: 2966–2977 16778770 10.1038/sj.emboj.7601184 PMC1500981

[embj2018101056-bib-0027] John GB , Shang Y , Li L , Renken C , Mannella CA , Selker JM , Rangell L , Bennett MJ , Zha J (2005) The mitochondrial inner membrane protein mitofilin controls cristae morphology. Mol Biol Cell 16: 1543–1554 15647377 10.1091/mbc.E04-08-0697 PMC551514

[embj2018101056-bib-0028] Kondadi AK , Anand R , Hansch S , Urbach J , Zobel T , Wolf DM , Segawa M , Liesa M , Shirihai OS , Weidtkamp‐Peters S *et al* (2019) Cristae undergo cycles of fusion and fission in MICOS‐dependent manner. bioRxiv [PREPRINT]. 10.1101/654541 PMC7054676 32067344

[embj2018101056-bib-0029] Leung CW , Hong Y , Hanske J , Zhao E , Chen S , Pletneva EV , Tang BZ (2014) Superior fluorescent probe for detection of cardiolipin. Anal Chem 86: 1263–1268 24372165 10.1021/ac403616c PMC3950812

[embj2018101056-bib-0030] Loew LM , Tuft RA , Carrington W , Fay FS (1993) Imaging in five dimensions: time‐dependent membrane potentials in individual mitochondria. Biophys J 65: 2396–2407 8312478 10.1016/S0006-3495(93)81318-3 PMC1225980

[embj2018101056-bib-0031] von der Malsburg K , Muller JM , Bohnert M , Oeljeklaus S , Kwiatkowska P , Becker T , Loniewska‐Lwowska A , Wiese S , Rao S , Milenkovic D *et al* (2011) Dual role of mitofilin in mitochondrial membrane organization and protein biogenesis. Dev Cell 21: 694–707 21944719 10.1016/j.devcel.2011.08.026

[embj2018101056-bib-0032] Mitchell P (1961) Coupling of phosphorylation to electron and hydrogen transfer by a chemi‐osmotic type of mechanism. Nature 191: 144–148 13771349 10.1038/191144a0

[embj2018101056-bib-0033] Mitchell P , Moyle J (1969) Estimation of membrane potential and pH difference across the cristae membrane of rat liver mitochondria. Eur J Biochem 7: 471–484 5776240 10.1111/j.1432-1033.1969.tb19633.x

[embj2018101056-bib-0034] Palade GE (1953) An electron microscope study of the mitochondrial structure. Anat Rec 114: 427–452 10.1177/1.4.188 13069686

[embj2018101056-bib-0035] Pham TD , Pham PQ , Li J , Letai AG , Wallace DC , Burke PJ (2016) Cristae remodeling causes acidification detected by integrated graphene sensor during mitochondrial outer membrane permeabilization. Sci Rep 6: 35907 27786282 10.1038/srep35907 PMC5081517

[embj2018101056-bib-0036] Presley AD , Fuller KM , Arriaga EA (2003) MitoTracker Green labeling of mitochondrial proteins and their subsequent analysis by capillary electrophoresis with laser‐induced fluorescence detection. J Chromatogr B 793: 141–150 10.1016/s1570-0232(03)00371-4 12880861

[embj2018101056-bib-0037] Rabl R , Soubannier V , Scholz R , Vogel F , Mendl N , Vasiljev‐Neumeyer A , Korner C , Jagasia R , Keil T , Baumeister W *et al* (2009) Formation of cristae and crista junctions in mitochondria depends on antagonism between Fcj1 and Su e/g. J Cell Biol 185: 1047–1063 19528297 10.1083/jcb.200811099 PMC2711607

[embj2018101056-bib-0038] Rampelt H , Zerbes RM , van der Laan M , Pfanner N (2017) Role of the mitochondrial contact site and cristae organizing system in membrane architecture and dynamics. Biochim Biophys Acta Mol Cell Res 1864: 737–746 27614134 10.1016/j.bbamcr.2016.05.020

[embj2018101056-bib-0039] Rieger B , Junge W , Busch KB (2014) Lateral pH gradient between OXPHOS complex IV and F(0)F(1) ATP‐synthase in folded mitochondrial membranes. Nat Commun 5: 3103 24476986 10.1038/ncomms4103

[embj2018101056-bib-0040] Skulachev VP (2001) Mitochondrial filaments and clusters as intracellular power‐transmitting cables. Trends Biochem Sci 26: 23–29 11165513 10.1016/s0968-0004(00)01735-7

[embj2018101056-bib-0041] Strauss M , Hofhaus G , Schroder RR , Kuhlbrandt W (2008) Dimer ribbons of ATP synthase shape the inner mitochondrial membrane. EMBO J 27: 1154–1160 18323778 10.1038/emboj.2008.35 PMC2323265

[embj2018101056-bib-0042] Twig G , Elorza A , Molina AJ , Mohamed H , Wikstrom JD , Walzer G , Stiles L , Haigh SE , Katz S , Las G *et al* (2008) Fission and selective fusion govern mitochondrial segregation and elimination by autophagy. EMBO J 27: 433–446 18200046 10.1038/sj.emboj.7601963 PMC2234339

[embj2018101056-bib-0043] Vogel F , Bornhovd C , Neupert W , Reichert AS (2006) Dynamic subcompartmentalization of the mitochondrial inner membrane. J Cell Biol 175: 237–247 17043137 10.1083/jcb.200605138 PMC2064565

[embj2018101056-bib-0044] Ward MW , Rego AC , Frenguelli BG , Nicholls DG (2000) Mitochondrial membrane potential and glutamate excitotoxicity in cultured cerebellar granule cells. J Neurosci 20: 7206–7219 10.1523/JNEUROSCI.20-19-07208.2000 PMC6772767 11007877

[embj2018101056-bib-0045] Wikstrom JD , Katzman SM , Mohamed H , Twig G , Graf SA , Heart E , Molina AJ , Corkey BE , de Vargas LM , Danial NN *et al* (2007) beta‐Cell mitochondria exhibit membrane potential heterogeneity that can be altered by stimulatory or toxic fuel levels. Diabetes 56: 2569–2578 17686943 10.2337/db06-0757

[embj2018101056-bib-0046] Wilkens V , Kohl W , Busch K (2013) Restricted diffusion of OXPHOS complexes in dynamic mitochondria delays their exchange between cristae and engenders a transitory mosaic distribution. J Cell Sci 126: 103–116 23038773 10.1242/jcs.108852

[embj2018101056-bib-0047] Wollweber F , von der Malsburg K , van der Laan M (2017) Mitochondrial contact site and cristae organizing system: a central player in membrane shaping and crosstalk. Biochim Biophys Acta Mol Cell Res 1864: 1481–1489 28526561 10.1016/j.bbamcr.2017.05.004

[embj2018101056-bib-0048] Zanna C , Ghelli A , Porcelli AM , Karbowski M , Youle RJ , Schimpf S , Wissinger B , Pinti M , Cossarizza A , Vidoni S *et al* (2008) OPA1 mutations associated with dominant optic atrophy impair oxidative phosphorylation and mitochondrial fusion. Brain 131: 352–367 18222991 10.1093/brain/awm335

[embj2018101056-bib-0049] Zerbes RM , Bohnert M , Stroud DA , von der Malsburg K , Kram A , Oeljeklaus S , Warscheid B , Becker T , Wiedemann N , Veenhuis M *et al* (2012) Role of MINOS in mitochondrial membrane architecture: cristae morphology and outer membrane interactions differentially depend on mitofilin domains. J Mol Biol 422: 183–191 22575891 10.1016/j.jmb.2012.05.004

[embj2018101056-bib-0050] Zhang J , Guan Z , Murphy AN , Wiley SE , Perkins GA , Worby CA , Engel JL , Heacock P , Nguyen OK , Wang JH *et al* (2011) Mitochondrial phosphatase PTPMT1 is essential for cardiolipin biosynthesis. Cell Metab 13: 690–700 21641550 10.1016/j.cmet.2011.04.007 PMC3119201

