# Supplementary material for: Individual cristae within the same mitochondrion display different membrane potentials and are functionally independent
Source: EMBO J. 2019 Oct 14;38(22):e101056. doi: 10.15252/embj.2018101056 (PMC6856616; doi:10.15252/embj.2018101056)
Supplement: Supplementary file 1 — Expanded View Figures PDF [file EMBJ-38-e101056-s001.pdf]

Expanded View Figures

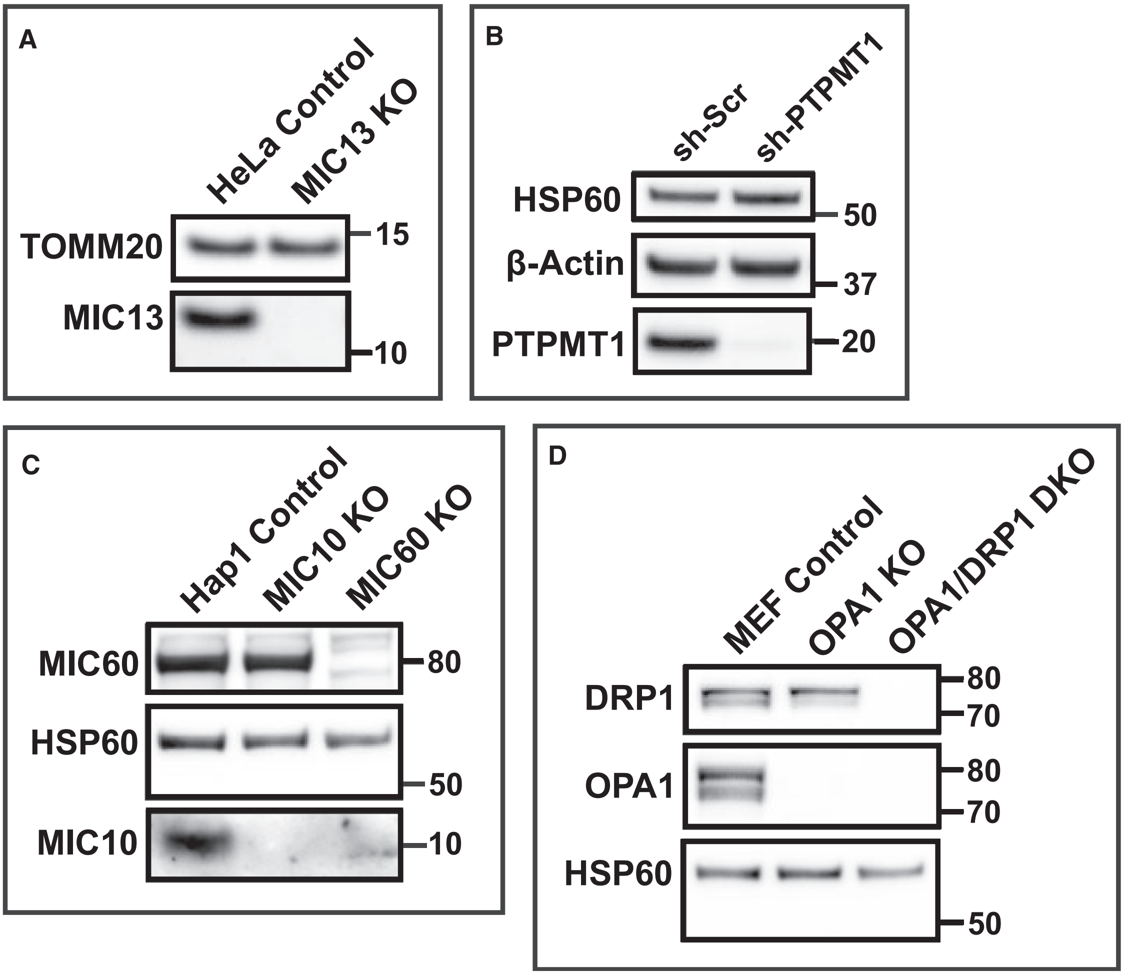

Figure EV1. Validation of KO and KD approaches of Cristae structure and CJ modulators.

A Representative image of Western blot, showing deletion of MIC13 in HeLa cells. *N* = 3 independent experiments.
B Representative image of Western blot, showing deletion of PTPMT1 in H1975 cells. *N* = 3 independent experiments.
C Representative image of Western blot, showing deletion of MIC60 and MIC10 in Hap1 cells. *N* = 3 independent experiments.
D Representative image of Western blot, showing Opa1 KO and Opa1&Drp1 DKO in MEFs. *N* = 3 independent experiments.

Source data are available online for this figure.

 © 2019 The Authors

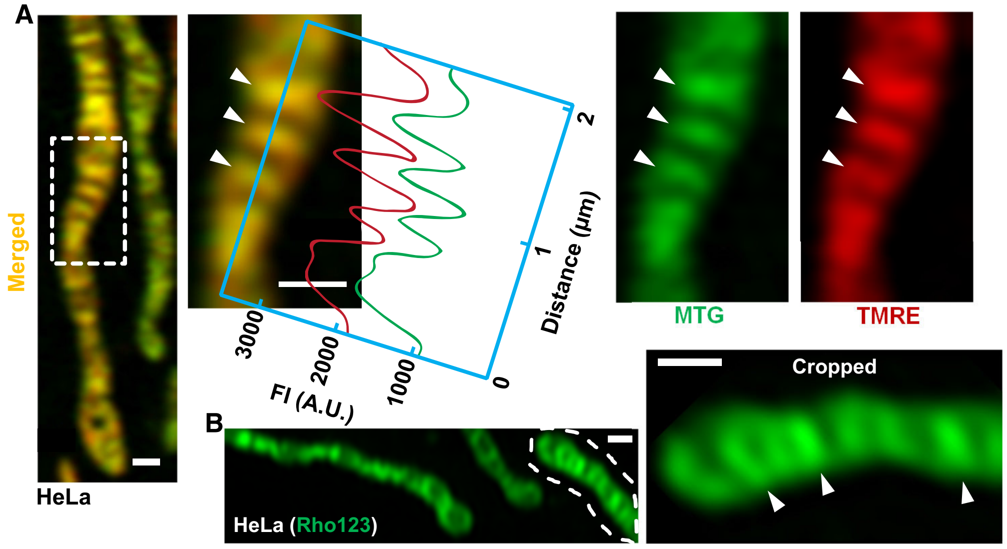

Figure EV2. Membrane potential dyes, TMRE and Rho123, are concentrated at the cristae membranes in a heterogeneous pattern.

A Mitochondria in HeLa cells co-stained with MTG and TMRE. Zoomed-in region from dashed white box highlights colocalization of dyes, showing that signal intensities vary together across the long axis (green and red lines); arrowheads indicate cristae membranes. Scale bar = 500 nm. *N* = 3 independent experiments.

B Mitochondria in HeLa cell stained with $\Delta\Psi_m$-dependent dye, Rho123; zoomed-in mitochondrion cropped from dashed line highlights more intense signal at cristae (arrowheads). Scale bar = 500 nm. *N* = 3 independent experiments.

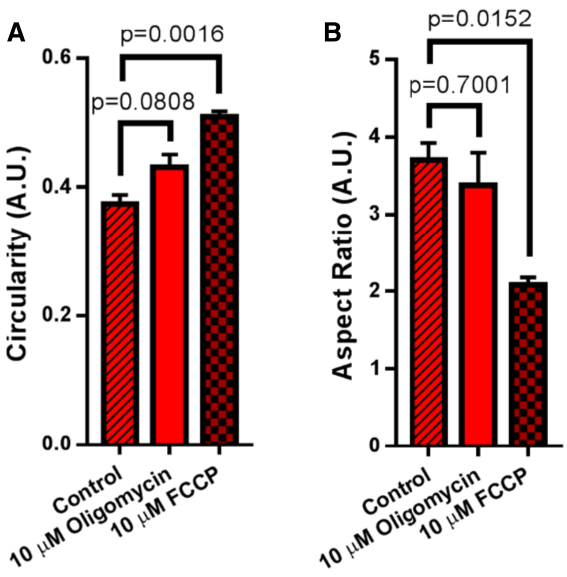

Figure EV3. Effects of FCCP and oligomycin on mitochondrial morphology.

A Quantification of mitochondrial circularity, where mitochondria with circularity closer to 1 resemble a circular object and mitochondria with circularity closer to 0 resemble a branched object. Note: FCCP results in significant increase in mitochondrial circularity, consistent with numerous studies indicating FCCP induces fragmentation. *N* = 3 independent experiments.

B Quantification of mitochondrial aspect ratio (a ratio of the long axis of the mitochondrion to the short axis), where mitochondria with an aspect ratio closer to 1 are rounder, whereas mitochondria higher and higher than 1 are increasingly elongated. Note: FCCP also leads to a significant decrease in aspect ratio, indicating loss of membrane potential results in fragmentation. *N* = 3 independent experiments.

Data information: Data were analyzed with 2-tailed Student's *t*-tests, and *P* values < 0.05 were considered statistically significant. Specific *P* values are indicated in the figure. Error bars indicate SEM.

© 2019 The Authors
