# Supplementary material for: Individual cristae within the same mitochondrion display different membrane potentials and are functionally independent
Source: EMBO J. 2019 Oct 14;38(22):e101056. doi: 10.15252/embj.2018101056 (PMC6856616; doi:10.15252/embj.2018101056)
Supplement: Supplementary file 2 — Movie EV1 [file EMBJ-38-e101056-s002.zip › Movie_EV1.docx]

**Movie Expanded View 1.**

Hyperpolarized vesicles moving haphazardly within the matrix of Opa1&Drp1-DKO mitochondria.
