# Supplementary material for: Individual cristae within the same mitochondrion display different membrane potentials and are functionally independent
Source: EMBO J. 2019 Oct 14;38(22):e101056. doi: 10.15252/embj.2018101056 (PMC6856616; doi:10.15252/embj.2018101056)
Supplement: Supplementary file 5 — Movie EV4 [file EMBJ-38-e101056-s005.zip › Movie_EV4.docx]

**Movie Expanded View 4.**

Mitochondrion from HeLa cell stained with MTG and TMRE, showing partial depolarization during random flickering event. Note: this movie shows the red (TMRE) channel only.
