# Supplementary material for: Individual cristae within the same mitochondrion display different membrane potentials and are functionally independent
Source: EMBO J. 2019 Oct 14;38(22):e101056. doi: 10.15252/embj.2018101056 (PMC6856616; doi:10.15252/embj.2018101056)
Supplement: Supplementary file 6 — Movie EV5 [file EMBJ-38-e101056-s006.zip › Movie_EV5.docx]

**Movie Expanded View 5.**

Mitochondrion from HeLa cell stained with MTG and TMRE, showing partial depolarization during random flickering event. Note: this movie shows the (TMRE) channel as an LUT to highlight the asymmetrical distribution of the ΔΨ_m_-dependent dye in the polarized (red) vs. depolarized (blue) regions.
