# Supplementary figures and images for: Individual cristae within the same mitochondrion display different membrane potentials and are functionally independent
Source: EMBO J. 2019 Oct 14;38(22):e101056. doi: 10.15252/embj.2018101056 (PMC6856616; doi:10.15252/embj.2018101056)

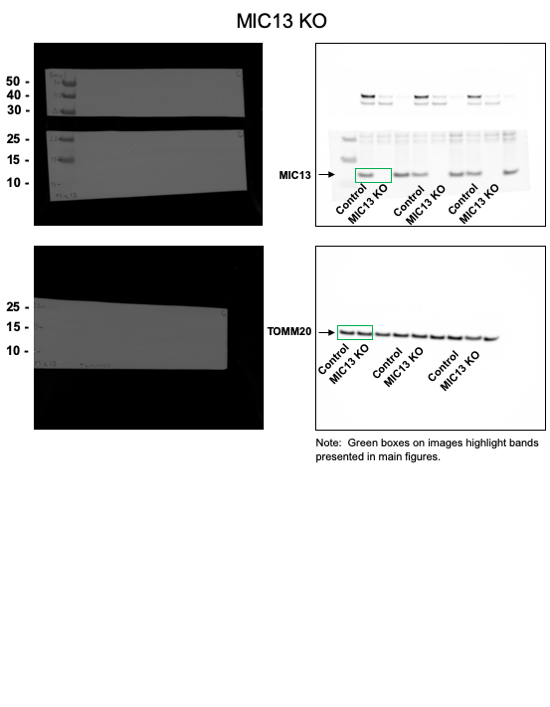

Supplement: Supplementary file 7 — Source Data for Expanded View [file EMBJ-38-e101056-s007.zip › Slide1.tiff]

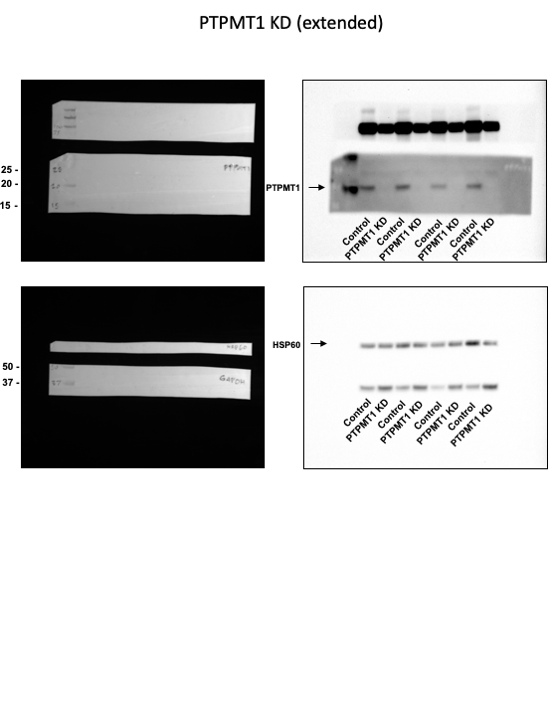

Supplement: Supplementary file 7 — Source Data for Expanded View [file EMBJ-38-e101056-s007.zip › Slide5.tiff]

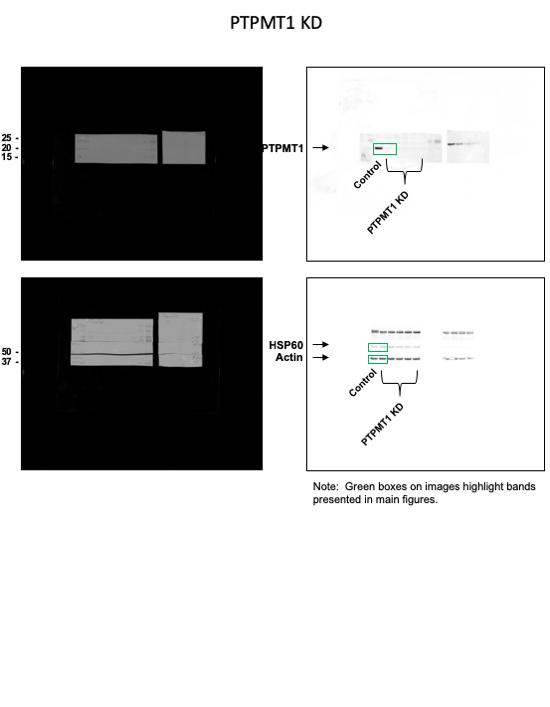

Supplement: Supplementary file 7 — Source Data for Expanded View [file EMBJ-38-e101056-s007.zip › Slide4.tiff]

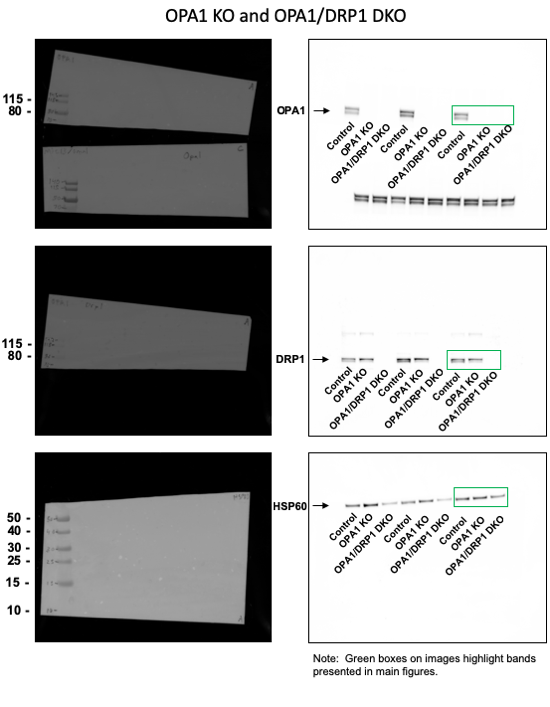

Supplement: Supplementary file 7 — Source Data for Expanded View [file EMBJ-38-e101056-s007.zip › Slide3.tiff]

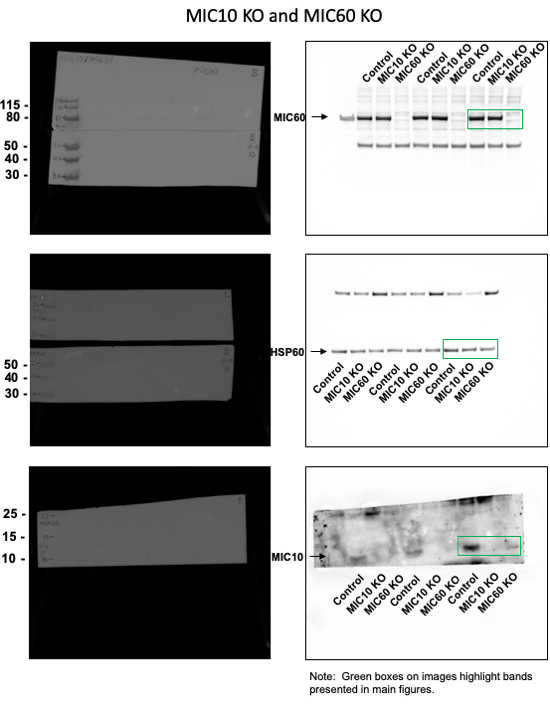

Supplement: Supplementary file 7 — Source Data for Expanded View [file EMBJ-38-e101056-s007.zip › Slide2.tiff]
